# Divergence and Selectivity of Expression-Coupled Histone Modifications in Budding Yeasts

**DOI:** 10.1371/journal.pone.0101538

**Published:** 2014-07-09

**Authors:** Yaron Mosesson, Yoav Voichek, Naama Barkai

**Affiliations:** Department of Molecular Genetics, Weizmann Institute of Science, Rehovot, Israel; CNRS, France

## Abstract

Various histone modifications are widely associated with gene expression, but their functional selectivity at individual genes remains to be characterized. Here, we identify widespread differences between genome-wide patterns of two prominent marks, H3K9ac and H3K4me3, in budding yeasts. As well as characteristic gene profiles, relative modification levels vary significantly amongst genes, irrespective of expression. Interestingly, we show that these differences couple to contrasting features: higher methylation to essential, periodically expressed, ‘DPN’ (*D*epleted *P*roximal *N*ucleosome) genes, and higher acetylation to non-essential, responsive, ‘OPN’ (*O*ccupied *P*roximal *N*ucleosome) genes. Thus, H3K4me3 may generally associate with expression stability, and H3K9ac, with variability. To evaluate this notion, we examine their association with expression divergence between the closely related species, *S. cerevisiae* and *S. paradoxus*. Although individually well conserved at orthologous genes, changes between modifications are mostly uncorrelated, indicating largely non-overlapping regulatory mechanisms. Notably, we find that inter-species differences in methylation, but not acetylation, are well correlated with expression changes, thereby proposing H3K4me3 as a candidate regulator of expression divergence. Taken together, our results suggest distinct evolutionary roles for expression-linked modifications, wherein H3K4me3 may contribute to stabilize average expression, whilst H3K9ac associates with more indirect aspects such as responsiveness.

## Introduction

A dynamic portfolio of gene transcripts shapes cellular phenotype, but much about the regulation of transcription remains to be understood. Clearly, multiple, convergent regulatory mechanisms, converge at individual genes, including recruitment of selective transcription factors (TFs), organization of nucleosomes, and post-translational modification of key components such as the RNA polymerase II complex (Pol II) and histone proteins. These mechanisms collectively govern different phases of the transcriptional cycle, such as initiation and elongation. Therefore, by assimilation of regulatory inputs, cells may construct different kinetic strategies of transcription, suited to the requirements of a particular gene [1].

Histone modifications, including acetylation, methylation, and ubiquitylation, have emerged as pivotal features of transcriptional regulation. Various forms, targeting specific histone residues, have been extensively associated with active gene expression in genome-wide studies throughout eukaryotes [2]–[6]. Acetylation levels are governed by the net activity of histone acetyl transferases (HATs) and histone deacetylases (HDACs). Prominent transcription-linked HATs, including Gcn5 and Esa1, selectively target lysine clusters mainly within the amino-terminal regions of histone 3 (H3) and histone 4 (H4). Gcn5, in turn, forms part of the SAGA complex, a multi-modular assembly extensively coupled to the transcription machinery [7]. Accordingly, SAGA is recruited to active promoters, at levels that reflect gene activity [8], and to coding regions, in patterns that mirror Pol II occupancy [9], [10]. Closely spaced acetyl marks are thought to promote the loosening or eviction of nucleosomes, both via collective neutralization of histone/DNA interactions [11], and by direct engagement of nucleosome remodeling complexes [12]. This facilitates binding of initiation factors at the promoter, and presumably, paves a path for Pol II progress across coding regions. Hence, histone acetylation is often synonymous with active transcription, and may influence the kinetics of both initiation and elongation. HDACs, in contrast, generally associate with repression. As with HATs, several HDACs, such as the prototypical Rpd3 complex, are enlisted at different regions along the gene body [13], [14], through various mechanisms, such as via direct engagement by various histone methyl marks, and recruitment by global repressor proteins (reviewed in [15]). Hence, by countering nucleosome pliability, deacetylation serves to prevent or retard transcriptional activity.

Histone methylation encompasses a variety of forms (mono-, di-, tri-, at several acceptor residues), and associates with both activated and repressed gene expression. Tri-methylation of H3K4 (H4K4me3), generated by Set1 methyltransferase, is most commonly correlated with active expression (reviewed in [16]). Set1, a component of the COMPASS complex, couples to several core regulators of transcription. In particular, COMPASS binding at active genes, and subsequent methylation, depends on mono-ubiquitylation of histone H2B (H2Bub) [17]. Indeed, both modifications are dependent on the Pol II-binding PAF complex, a global regulator that serves a platform for various histone-modifying activities during Pol II progression [18]. These associations implicate H2Bub and H3K4me 3 (as well as other methyl marks) in the control of elongation. As well as a methylation-deacetylation cross talk, H3K4me3 marks may also promote acetylation via methyl-binding domains within components of HAT complexes, including SAGA [19]. SAGA also incorporates a de-ubiquitylation module targeting H2Bub [20]. On aggregate, therefore, histone modifications are richly inter-linked both positively and negatively, in line with a model wherein orchestrated turnover of histone marks facilitates the disassembly and reassembly of nucleosomes during Pol II passage.

Interactions between histone-modifying enzymes raise an important issue; namely, to what degree are their corresponding marks connected in the context of expression? Are these generically utilized within a transcriptional cycle, or are their distinct chemical attributes exploited in order to forge customized kinetic programs? An extensive regulatory repertoire associated with each modification suggests that, in evolutionary terms, a degree of functional independence will have been encoded. Notably, by deletion of individual chromatin regulators in budding yeast, only limited changes in steady-state expression were observed, although functional inter-relationships between regulators could be reconstructed based on their gene targets [21]. This indicates the likely importance inter-complex cooperativity, but also attests to gene selectivity of regulatory complexes.

To investigate the selective nature of histone marks, we focus on the relationship between H3K9ac (generally representative of Gcn5-dependent acetylation of several other lysines within H3) and H3K4me3 in budding yeasts. Two complementary approaches are taken: firstly, we compare genome-wide profiles of both modifications in *S. cerevisiae*, and analyze their concordance at individual genes. We ask: can we distinguish different modification patterns with respect to nucleosome and gene preferences? Can we infer functional attributes from differences in gene targets? Second, we explore divergence of H3K9ac and H3K4me3 between the closely related species, *S. cerevisiae* and *S. paradoxus*. This approach offers a valuable means to evaluate the evolutionary association between sets of parameters, and therefore the degree to which these share a common genetic basis, and in turn, mechanisms of regulation. Hence, it allows us to interrogate the level of inter-dependence between H3K9ac and H3K4me3. Likewise, we ask how divergence of each modification correlates with expression divergence, and therefore whether or not either may serve as an evolutionary candidate to modulate expression changes between related species. Previous association studies in different yeast strains suggest that a large fraction of expression differences may be linked to polymorphisms affecting chromatin regulators [22], [23]. On the other hand, features such as nucleosome occupancy and TF binding sites may only weakly explain expression divergence between species [24], [25]. Hence, we evaluate the evolutionary utility of these histone marks in this context.

Our analyses reveal widespread differences between acetylation and methylation. Interestingly, each mark is found enriched at contrasting types of genes: higher H3K9ac at genes often variably transcribed (non-essential, responsive and OPN genes), and residual H3K4me3 at those more rigidly expressed (essential, periodic and DPN genes). Comparisons between species suggest largely independent regulation of each modification, and moreover, reveal significantly better agreement of methylation and expression divergence. Taken together, our results indicate that selective utilization of these chromatin features in different transcriptional programs may be highly prevalent, and further, propose that acetylation engenders variable transcription while H3K4me3 contributes to a more stable program.

## Materials and Methods

### Yeast strains and antibodies

*S. cerevisiae* (BY4743) and a homozygous diploid *S. paradoxus* (generated from the CBS432 strain by transient HO inactivation) were used. ChIP antibodies to acetyl K9 of histone H3 (anti-H3K9ac), tri-methyl K4 of histone H3 (anti-H3K4me3), and an unmodified histone H3 were from Abcam (ab4441, ab8580, and ab1791, respectively).

### Chromatin immunoprecipitation and mRNA extraction

ChIP was performed as previously described (Weinberger et al. 2012). Briefly, cells growing at mid-log phase (OD∼0.7) were fixed, lysed and sonicated to generate DNA fragments with average size of 150-300 bp. Antibodies were added for an overnight incubation and precipitated using Protein A agarose beads. Following extensive washes, antibody-bound DNA was eluted in SDS and cross-links were reversed by incubation at 65°C. Proteins and RNA were then degraded, and the DNA obtained using the Qiagen PCR purification kit. To verify specific enrichment of DNA, multiplex PCR was performed at several loci selected according to previously published data (Liu et al. 2005). mRNA was generated in parallel from aliquots taken prior to cell fixation. Equal amounts of polyA-containing RNA from *S. cerevisiae* and *S. paradoxus* were taken before reverse transcription and library preparation for high throughput sequencing.

### ChIP-seq and RNA-seq analysis

High-throughput sequencing was performed using the Illumina GAIIx system. To minimize experimental artifacts between samples, ChIP samples using the same antibody in the two yeast species were combined and processed together. Likewise, RNA samples were also combined. Raw data (40 base reads) were aligned to reference genomes of both *S. cerevisiae* (from Saccharomyces Genome Database) and *S. paradoxus* (obtained from the SGRP at the Sanger Institute), using the Illumina pipeline (Casava) together with Bowtie software [26]. Given significant sequence divergence between the species (∼10% non-identical), almost all reads (>95%) could be unequivocally matched to either one or the other reference. To more accurately score each base, we estimated the characteristic length of the DNA fragments for each sequenced sample. This was done by generating separate profiles for the forward and reverse strands (scoring the first sequenced base), shifting the profile of one strand a base at a time, and calculating the overall correlation of the shifted profile with that of the other strand. The offset giving maximal correlation was considered as the average fragment length (typically, c. 125 bp, with rank correlation between strands of ∼0.70). To obtain a profile per base pair, the forward and reverse strand profiles were shifted towards each other (by half the optimal offset) and then summed. Because the individual samples did not yield the same total number of reads, all profiles were normalized to that with the lowest total (∼6.5 million reads).

For individual genes, we took the modification profile around the transcription start site (TSS) including the promoter region (−500 bp to +900 bp relative to the TSS), and that around the transcription termination site (TTS; −400 bp to +200 bp relative to the TTS). Binned profiles per gene around these regions were then obtained by taking the log_2_-transformed mean signal over 20 bp intervals. In order to derive modification levels at individual nucleosomes in *S. cerevisiae*, we utilized consensus coordinates in assembled by Jiang et al. from several high-resolution maps of nucleosome occupancy (Jiang and Pugh 2009). Here, nucleosomes per gene were also numbered according to their position relative to the largely consensual TSS nucleosome: −2 and −1 for promoter-residing nucleosomes, +1 for the TSS nucleosome, and +2, +3, etc., for successive downstream nucleosomes (Jiang and Pugh 2009). Mean modification levels encompassing individual nucleosomes were taken and log_2_ transformed. For nucleosome occupancy profiles around the TSS and TTS, we employed previously generated MNase-seq data for *S. cerevisiae* and *S. paradoxus* from our laboratory [24]. Sequenced reads were binned as above. For RNA-seq experiments, reads were mapped to the reference genomes and the number of reads residing between the gene TSS and TTS summed. Gene expression levels were determined by normalizing for gene length and for the total number of reads mapped, and after taking an average of two biological repeats.

Because the *S. paradoxus* reference genome was incompletely annotated, we localized the orthologous genes by aligning the *S. cerevisiae* gene sequences with the appropriate chromosome of *S. paradoxus*. Most of genes (c. 90%) were aligned, and the orthologous TSS and TTS coordinates identified. Thereafter, binned modification profiles around the TSS and TTS were obtained as above. To facilitate interspecies comparisons, we also extracted average modification levels at three regions along the gene: the promoter (denoted ‘prom’: −320 to −160 relative to the TSS), the regions with highest abundance for each modification (denoted ‘peak’: 0 to +140 for H3K9ac, +100 to +580 for H3K4me3) and the end of genes (denoted ‘end’: −260 to +60 relative to the TTS). Furthermore, for interspecies comparisons at these regions, genes lacking data for either one of the modifications, mostly corresponding to short genes, were excluded from the analysis (e.g. leaving c. 4900 genes for analysis of ‘peak’ H3K9ac and H3K4me3).

### Normalization of H3K9ac and H3K4me3 ChIP-seq data for Histone H3

In order to normalize H3K9ac and H3K4me3 levels in *S. cerevisiae* for nucleosome occupancy, we performed ChIP-seq using an anti-H3 antibody. The same experimental protocol described was employed, including similar growth conditions, sonication procedure, and Illumina pipeline. We obtained a lower number of mapped sequencing reads (∼2.2 million) due to lower antibody efficacy. H3 levels were smoothed using a moving window (of 140 bases), and profiles per gene then generated as above, taking the mean signal over 20 bp intervals. For normalization, we subtracted H3 from modification levels (log_2_-transformed), either at each binned position along a gene, or after taking mean modification and H3 levels, either across the respective ‘peak’ regions or across the proximal open reading frame (−60 to +580 relative to the TSS).

### Gene classifications and statistical analyses

Classifications of DPN and OPN genes were taken from a previous study [27]. Likewise, annotation of TATA and TATA-less genes was from previous work [28]. For periodic genes, we utilized a data set generated in Pramila et al., comprising the probabilities for genes to be periodically transcribed, which were calculated from measurements of transcript levels in synchronized cells at high temporal resolution [29]. We took the top 800 ranked genes for our analysis. A measure of expression responsiveness was calculated from a compendium of >1500 expression data sets for all genes, under a variety of conditions, including environmental stresses, mutations and developmental transitions [30]. We took the standard deviation of expression per gene as a measure of responsiveness: the top and bottom ranked genes were assigned as responsive and non-responsive, respectively (1000 genes each). For classification according to gene ontology, we used the GO slim annotations, comprising 83 groupings (www.geneontology.org). Genes were also grouped according their tendency to be co-expressed with other genes; here, we employed previous data from our lab, comprising 26 pre-defined, overlapping transcriptional modules, which were constructed thorugh analysis of numerous expression data sets [30]. Enrichment or depletion was calculated via a hypergeometric test in Matlab, or as a percentage of the abundance expected for the null hypothesis.

## Results

### Distinct genomic patterns of H3K9ac and H3K4me3 in *Saccharomyces cerevisiae*

**Figure 1:**
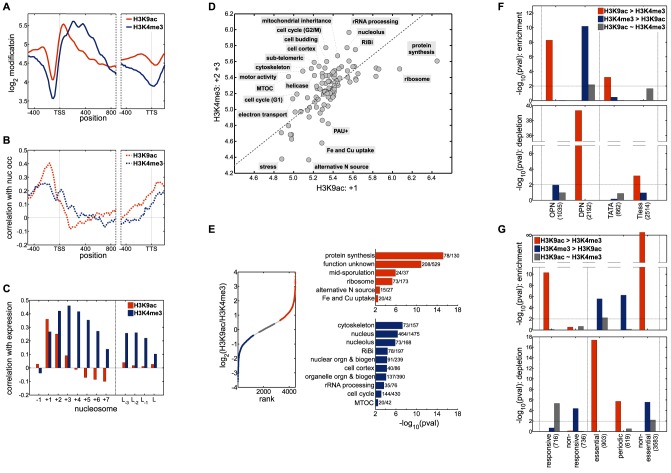
Biased distributions of expression-associated histone modifications in *S. cerevisiae*. **(A)** Average H3K9ac (*red*) and H3K4me3 (*blue*) profiles around the transcription start and termination sites of genes (*TSS* and *TTS*, respectively). **(B)** Traces depicting the correlation of H3K9ac and H3K4me3 profiles with nucleosome occupancy, around the TSS and TTS of genes. **(C)** Bar graphs showing the correlation between mRNA expression and modifications at individual nucleosomes across genes, as indicated. **(D)** Levels of H3K9ac (at +1nuc) and H3K4me3 (mean of +2nuc and +3nuc levels) were obtained for all genes. Genes were then grouped by gene ontology (‘GO slim’ categories, www.geneontology.org) or by pre-defined transcriptional modules [30], and the mean H3K9ac and H3K4me3 per category plotted against each other. Gene groups exhibiting higher average levels of one modification over the other are indicated. **(E)** Modification levels at specific nucleosomes for all genes were taken as in (D), and the ratio between H3K9ac and H3K4me3 calculated (log_2_(H3K9ac/H3K9ac)). Genes were ranked according to this ratio, and three sectors (1200 genes each) were considered: H3K4me3 > H3K9ac (*blue*), H3K4me3 ≈ H3K9ac (*grey*), and H3K4me3 < H3K9ac (*red*). Enrichment of various categories of genes (as in (D)) within each of these sectors was then assessed using a hypergeometric test. Significantly enriched categories (−1*log_10_(pval) > 2) are depicted in the bar graphs. **(F)** Genes were classified according to their promoter nucleosome architecture (occupied proximal nucleosome, *‘OPN’*; depleted proximal nucleosome, *‘DPN’*), or according to whether or not they incorporate a TATA-box within the promoter (TATA-containing, *‘TATA’*; or TATA-deficient, *‘Tless’*). Thereafter, enrichment (*top panel*) or depletion (*bottom panel*) of these classes amongst the sectors defined in terms of the genic H3K9ac/H3K4me3 ratio (as in (E)) was calculated using a hypergeometric test (*top panel)*. Calculated p values are shown as -1*log10(pval)). The number of genes in each subgroup is indicated. **(G)** As in (F) but assessing the enrichment (*top panel*) and depletion (*bottom panel*) of genes classified according to several features of expression: ‘*responsive*’ and ‘*non-responsive*’ genes, defined by their expression variance across a large compendium of conditions [30]; ‘*periodic*’ genes, which show cyclical expression between successive cell cycles (800 genes; as defined in [29]); ‘*essential*’ and ‘*non-essential*’ genes (defined according to the viability in rich media of their respective deletion mutants).

Despite common ties to gene transcription, the extent to which H3K9ac and H3K4me3 patterns coincide within the genome remains unclear. To address this, we generated high-resolution maps in *S. cerevisiae*: cells growing in rich conditions (YPD) were subjected to chromatin immunoprecipitation (ChIP) with specific antibodies and subsequent high-throughput sequencing using the Illumina platform (see Materials and Methods for details). Sequenced reads were mapped, and to facilitate comparison between modifications, normalized for total read counts and log_2_ transformed. For a general perspective, we assessed their relative abundance at gene promoters and at open reading frames (ORFs; taking the mean signal across these regions). As shown, acetylation is clearly more enriched at gene promoters compared to methylation (Figure S1A in File SI), suggesting their differential function within this region. Both modifications are similarly prevalent within the ORF. We then plotted average modification profiles after aligning genes by their transcription start or end sites (TSS and TTS, respectively). As depicted, H3K9ac peaks at the TSS and sharply declines at downstream nucleosomes, while H3K4me3 exhibits a broader plateau encompassing several nucleosomes within the proximal ORF, which decreases thereafter (Figure 1A). These distinct profiles in *S. cerevisiae* are in accord with recent ChIP-seq studies [14], [31], [32]. Employing consensus genome-wide nucleosome positions compiled from several high-resolution maps [33], we inquired on modification patterns at individual nucleosomes. Here, nucleosomes are numbered according to their location along a gene: ‘-2nuc’ and ‘-1nuc’ encompass promoter-residing nucleosomes, ‘+1nuc’ generally lies at the TSS, and nucleosomes further downstream are denoted ‘+2nuc’, ‘+3nuc’, etc. As shown, distributions for successive nucleosomes confirm highest abundance of H3K9ac marks at +1nuc, and that a significant fraction of genes also show high acetylation at -1nuc, while H3K4me3 is most prevalent at +2nuc and +3nuc (Figure S1B in File SI).

Given these differences, we assessed the influence of nucleosome occupancy in shaping the distinct profiles. To address this, average acetylation and methylation at individual nucleosomes were plotted after sorting by increasing occupancies. Interestingly, both modifications at -1nuc tended to mirror occupancy, while no association could be observed at nucleosomes that harbor highest H3K9ac and H3K4me3 levels (Figure S1C in File SI); accordingly, a positive correlation between modification and occupancy levels within the promoter (r_ = _0.2 to 0.4) declines to zero within the transcribed region (Figure 1B). This indicates that profiles within the gene body are actively determined, either by depositing/removing enzymes or as a consequence of nucleosome turnover, whereas regulation of promoter modification levels may be less dynamic.

In order to directly account for the contribution of nucleosome occupancy, we performed ChIP-seq using an antibody to Histone H3, employing the same conditions as above. We then normalized for occupancy by subtracting log-transformed H3 from modification levels for each position (see Materials and Methods). Examples of H3K9ac and H3K4me3 profiles at individual genes before and after H3 normalization are depicted (Figure S2A in File SI). To gauge the general effect of occupancy, we plotted the average H3-normalized modification levels along a gene. As shown, the characteristic modification profiles are similar after normalizing for H3 levels: H3K9ac clearly peaks at the TSS nucleosome, is also prevalent at the promoter, and sharply diminishes at internal ORF nucleosomes; H3K4me3 remains maximal at +2nuc and +3nuc (Figure S2B in File SI). Hence the distinct nucleosome preferences for these modifications are underscored after taking nucleosome occupancy into consideration.

Within this context, the positional association of modifications with gene expression was assessed. We generated RNA-seq data in cells growing under similar conditions, and expression levels were assigned by averaging mapped read counts across a gene. As expected, after classifying genes by expression level, average H3K9ac and H3K4me3 profiles unravel accordingly; that is, genic peak heights directly reflect the median expression of respective groups (Figure S1D in File SI). Examined per nucleosome, it is clear that association with expression varies along a gene: maximal correlation with H3K9ac occurs at +1nuc (r≈0.35), and with H3K4me3, at +2nuc to +4nuc (r≈0.45; Figure 1C), in accord with previous results [4], [14], [31]. Respectively, these comprise the nucleosomes with highest signals, which are also independent of occupancy. While promoter modifications showed no correlation with expression, we found a weak negative association with acetylation at distal coding-region nucleosomes, perhaps due to active de-acetylation. To corroborate these results, we also tested expression correlation after taking nucleosome occupancy into account. As above, average H3-normalized H3K9ac and H3K4me3 profiles for genes grouped by expression were well separated according to the group’s mean expression level (Figure S2C in File SI, upper and middle panels). Further, the different positional correlations across a gene for H3K9ac and H3K4me3 were also maintained (Figure S2C in File SI, lower panel).

In summary, although mutually coupled to expression, H3K9ac and H3K4me3 concentrate to different nucleosomes along a gene. Conceivably, this may demarcate distinct functional domains during transcription: enrichment of acetylation at the TSS may drive initiation [34], while broader downstream methylation could serve a role in the fidelity of incipient transcription [35]. We note that these steady state profiles are the net outcome of extensive crosstalk between multiple histone modifying enzymes, hence, dynamic modification at other nucleosomes may be highly significant. Still, the question arises as to whether or not H3K9ac and H3K4me3 are similarly invoked at a particular gene during transcription.

### Disparity between H3K9ac and H3K4me3 levels prevails at specific classes of genes

Mutual links to gene expression ensure a minimal coordination between acetylation and methylation, but to what degree does this occur? According to one possibility, if both marks contribute equivalently within a transcriptional cycle, then one might expect synchrony at all genes. Alternatively, these marks may have been deployed unevenly, for instance, in order to exploit different activities that each presents. To address this, intragenic modification levels were compared. Here, we examined only those nucleosomes that best correlate with expression, as we considered that incorporating other regions would likely detract from focusing on expression-linked phenomena. Hence, taking H3K9ac at +1nuc and average H3K4me3 across +2nuc and +3nuc, we calculate a Pearson correlation of ∼0.3 (Figure S1E in File SI, left panel), which indicates that for given expression, relative modification levels may fluctuate significantly. Furthermore, after normalizing for expression, the correlation between modifications that can be attributed to expression is weaker (Figure S1E in File SI, right panel; r≈0.3–0.15). Hence, in the context of expression, the perceived lack of coordination may either reflect redundant functions of these modifications, or else be concealing deliberate asymmetry of acetylation-vs-methylation at a particular gene.

To examine this issue, we surveyed the gene pool by drawing on pre-assembled categories: 83 groups based on gene ontologies (GO slim database; www.geneontology.org), and an additional 26 groups comprising transcriptional modules re-constructed from multiple expression data sets [30]. Average acetylation and methylation per group were then compared. As shown, most display similar relative levels, indeed reflecting a much higher correlation coefficient compared to that for individual genes (∼0.65; Figure 1D). Predictably, gene sets that are highly/poorly expressed in rich media contain high/low amounts of both marks on average (e.g. ‘Golgi apparatus’ or ‘cytoplasmic vesicle’ vs ‘peroxisome’ or ‘meiosis’, respectively). We estimated the fraction of individual genes showing concordant modification levels, by taking the log_2_(H3K9ac/H3K4me3) ratio and defining a nominal cutoff for modification differences of ±0.5 from the median (∼ 1.4-fold; data not shown). By these criteria, c. 45% of genes exhibit similar modification levels, corresponding to the indicated correlation coefficient of ∼0.3 (Figure S1E in File SI). For a minority of groups, however, disparity between H3K9ac and H3K4me3 persists across the constituent genes. For instance, on average, ‘protein synthesis’ genes appear hyper-acetylated, while ‘rRNA processing’ genes are, on average, methylation-rich (Figure 1D), suggesting that these biases may result from shared regulatory features at such genes.

To adopt a more systematic approach, we calculated the ratio of modifications (log_2_ (H3K9ac/H3K4me3)) at individual genes, and then ranked them accordingly. For this analysis, we considered genes above a minimal gene length (700 bases; ∼4500 genes); because methylation, on average, concentrates at several nucleosomes within the proximal ORF, we reasoned that this constraint allows a more suitable comparison between modifications per gene. Three classes (1200 genes each) were then taken - the highest (H3K9ac > H3K4me3) and lowest (H3K4me3 > H3K9ac) ranked, and those intermediately ranked (H3K9ac ≈ H3K4me3) - and subsequently analyzed for enrichment of our pre-defined categories. Indeed, genes engaged in protein synthesis, in particular, were highly enriched within the first class (hypergeometric test, p≈10^−15^), while higher methylation was significant for cytoskeletal (p≈10^−7^) and nucleolar genes (p≈10^−6^), as well as genes involved in biogenesis/assembly of ribosomes (‘RiBi’), organelles and the nucleus (p≈10^−4^; Figure 1E). Examples of gene contingents with tendencies towards with a higher acetylation or methylation are shown (Figure S1F in File SI). No categories were found to preferentially contain concordant acetylation and methylation levels.

A possible caveat of the analysis relates to the comparison of modifications at different nucleosomes along a gene (+1nuc versus +2/3nucs). As described, these regions were taken because we reasoned these to be the most informative in the context of active expression, based on their different positional correlations with expression (Figure 1C). However, variations in nucleosome occupancies at these positions may directly impact on relative H3K9ac and H3K4me3 amounts at individual genes, and so affect our analysis. Hence, to address this issue, we employed H3-normalized modification levels at these positions. Here, we took the mean log_2_-transformed H3 and modification signal across the respective regions (−60 to +140 for H3K9ac, +100 to +580 for H3K4me3, relative to the TSS; Figure S3A in File SI), and subtracted H3 from modification values. Genes were then ranked, and enrichment of gene groups tested assessed as before. As shown, similar gene ontologies recurred in this more stringent analysis, albeit manifesting with generally lower p-values (Figure S3B in File SI); for example, higher H3K9ac remained significant for protein synthesis and ribosome genes (p≈10^−10^ and 10^−5^, respectively), and higher H3K4me3 for nucleolar and cytoskeletal genes (p≈10^−6^ and 10^−5^, respectively). Another possible issue is that prevalence of modifications across individual nucleosomes may vary significantly from gene to gene, notwithstanding the characteristic average profiles. Accordingly, we also considered a less stringent definition, wherein we took a broader region encompassing the proximal ORF (−60 to +580, essentially nucleosomes +1 to +4) for both H3K9ac and H3K4me3 (Figure S4A in File SI). For this analysis, we tested H3-normalized, as well as the original modification data. Indeed, both definitions yielded similar results, and strongly supported previous observations; recurrent gene ontologies were enriched for differential modification, and further, additional significant groups were apparent amongst the higher H3K4me3 category (e.g. ‘vesicle-mediated transport’, ‘helicase activity’; Figure S4B in File SI, and data not shown). Overall, by accounting for nucleosome occupancy, and by taking a broader definition of H3K9ac and H3K4me3 levels, modification biases amongst particular gene groupings are reaffirmed.

These observations suggest unique functional properties of each modification. Therefore, we asked whether or not their disparity associates with structural and other attributes of genes. Patterns of promoter nucleosome occupancy have been strongly implicated in differential regulation of transcription. Genes with nucleosomes positioned over their promoter region (‘occupied proximal nucleosome’; OPN), often occluding TF binding sites, tend to exhibit ‘plasticity’ of expression between cellular states. This includes non-uniform expression within an isogenic population, expression responsiveness to environmental changes, and divergent expression between related species [27], [36]. Presumably, the opportunity for such variability stems from the need to mobilize a multifaceted regulatory program, which includes de-occlusion of TF binding sites, in order for transcription to ensue productively. At the other end are genes characterized by nucleosome-free promoter regions, flanked by well-positioned nucleosomes (‘depleted proximal nucleosome’; DPN). This design likely facilitates assembly of the general transcription machinery, and may reflect a more homogeneous, less sensitive regulatory regime. Likewise, promoters that incorporate a TATA-box are often found in variable genes, while TATA-less genes are usually less variable [28]. Interestingly, we found that these contrasting features revealed clear preferences: OPN genes were significantly enriched in genes with higher H3K9ac (hypergeometric test, p≈10^−8^; fold enrichment over the expected abundance (fe) ≈5%), and DPN genes were more prevalent in the class defined by higher H3K4me3 (p≈10^−10^; fe≈15%) and markedly under-represented in the higher H3K9ac class (p≈10^−38^; fe≈−25%; Figure 1F, and data not shown). Despite considerable overlap with DPN genes, TATA-less genes were not enriched in any class, while TATA-containing genes showed similar, but less significant, tendencies as OPN genes. Notably, these observations are well corroborated by further analyses employing H3-normalized modification levels. As shown, the corresponding enrichment/depletion of OPN and DPN genes is maintained, while TATA status appears not to be linked (Figure S3C in File SI, left panels). Moreover, when taking average H3K9ac and H3K4me3 levels over the proximal ORF, as before, the modification preferences at OPN and DPN genes are generally strengthened (e.g. fe≈45% and fe≈25%, for higher H3K9ac and higher H3K4me3, respectively; Figure S4C in File SI, left panels). Taken together, these results suggest that nucleosome architecture may play a role in selective engagement of histone marks.

Other aspects of gene expression were also analyzed within this context, namely, responsiveness/plasticity and periodicity. ‘Responsiveness’ describes the potential expression range available to particular gene, and therefore, an indication of its sensitivity to regulatory inputs. To quantify responsiveness, we employed a compendium of transcription profiles at all genes in a variety of conditions (>1500 conditions, including environmental stresses, mutations and developmental transitions [30], and calculated for each gene the average magnitude of expression modulation. Periodicity, on the other hand, specifies the regularity with which genes are expressed between successive cell cycles; presumably, certain classes of genes, such as those with a ‘housekeeping’ role, encode an ability to be consistently and robustly transcribed. Highly periodic/cyclical genes were taken from previous studies employing finely resolved temporal expression profiles in synchronized cells [29]. In a similar vein, essential and non-essential genes were also analyzed. Clear differences also emerged on the basis of these classifications: higher acetylation relative to methylation was highly favored for responsive (p≈10^−10^; fe≈35%) and for non-essential genes (p > 10^−100^; fe≈10%; Figure 1G, and data not shown). Essential and periodically expressed genes, on the other hand, frequently displayed higher methylation (p≈10^−5^ and 10^−6^; fe≈25% and 30%, respectively), and furthermore, clearly disfavored higher acetylation (respectively, p≈10^−17^ and 10^−6^; fe≈−40% and 25%; Figure 1G, and data not shown). As previously, we re-appraised these findings using the alternative measures for modification levels described (H3-normalized, and mean over the proximal ORF). Here, preferences for differential acetylation-to-methylation clearly recapitulated for essential and non-essential genes, but responsiveness was only significant in the broader analysis (Figure S3C and Figure S4C in File SI, right panels).

To test the relative influence these parameters, we asked how their combination with nucleosome architecture affects the association with modification disparity. As shown, OPN/DPN status was generally a major determinant, as the respective biases were mostly retained amongst various sub-groups (Figure S5A in File SI, and data not shown). Periodicity and essentiality, however, associated with higher methylation, appeared to over-ride the preference of OPN genes for higher acetylation, which suggests that other regulatory features in addition to nucleosome architecture may guide selective use of modifications. Responsiveness *per se*, although initially associated with higher H3K9ac, did not affect the H3K4me3 bias at DPN or essential genes (Figure S5A in File SI, and data not shown), perhaps indicating the importance of H3K4me3-linked mechanisms at such genes. In further examples, we re-examined candidate GO categories (ribosome, RiBi, cytoskeletal and nuclear genes, and genes with unknown function). As shown, despite a tendency to fall within particular modification classes, further sub-division on the basis of OPN/DPN status augmented or diminished their enrichment accordingly, again underscoring the influence of nucleosome structure (Figure S5B in File SI).

Taken together, these results raise an interesting hypothesis; namely, that expression-linked histone modifications are not equivalently or redundantly used, but rather may be mobilized in a selective manner to create suitable programs of transcription. Higher H3K4me3, recapitulated at essential, periodic and DPN genes, may encompass a ‘stable’ strategy, wherein H3K4me3 guides reliable gene transcription to pre-programmed levels, without necessarily disclosing the temporal itinerary. Higher H3K9ac marks at intrinsically variable OPN genes may embody a more dynamic strategy; conceivably, residual steady-state TSS acetylation reflects rapid initiation of transcription, serving to meet changeable expression targets on demand (see Discussion).

### Divergence of H3K9ac and H3K4me3 patterns in related yeasts

Our observations suggest that each modification associates with a distinct feature of transcriptional regulation. In order to test our hypothesis, we adopted an evolutionary perspective, and analyzed divergence patterns amongst related yeast species. This offers two advantages: first, by evaluating the evolutionary coordination between acetyl and methyl marks, we may question whether or not their respective machineries are governed by common genetic ground, and therefore, the likelihood or not that they are functionally autonomous. Second, a similar analysis with expression divergence discloses the extent to which genetic regulation of transcription encompasses that of each histone mark. A strong evolutionary relationship would indicate a direct functional association (either causal or consequential) with average expression levels, while lack of correlation would point to an indirect role, and/or links to processes other than gene expression. Hence, if we propose that H3K4me3 is involved in the stabilizing expression between successive cell generations, then one might expect good agreement with expression divergence between evolved states. Conversely, with a putative role in the kinetics of transcription rather than yield, weaker evolutionary coordination of H3K9ac changes might be anticipated.

**Figure 2:**
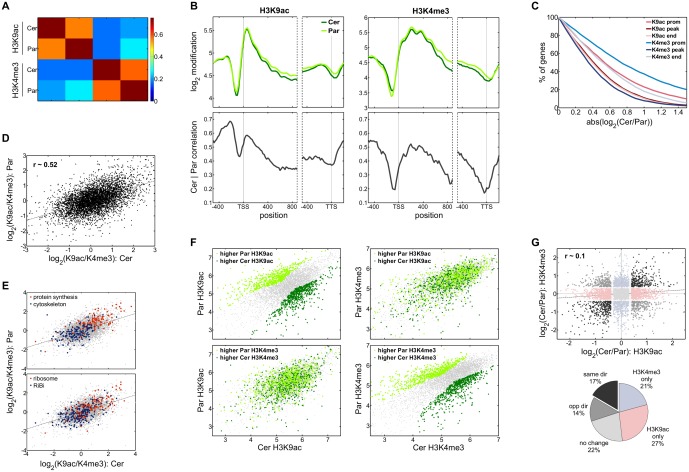
Divergence of H3K9ac or H3K4me3 patterns between closely related yeasts. **(A)** Overall correlation of H3K9ac and H3K4me3 patterns between *S. cerevisiae* and *S. paradoxus*. Heatmap of Pearson correlations calculated after juxtaposing modification profiles (mean signal over 20bp intervals) at c.6000 orthologous genes (including the promoter and coding regions). **(B)** Average H3K9ac (*upper left panel*) and H3K4me3 (*upper right panel*) profiles around the TSS and TTS, for *S. cerevisiae* (*dark green*) and *S. paradoxus* (*light green*). Interspecies correlations for H3K9ac and H3K4me3 at the given positions along a gene are shown in the lower panels. **(C)** Interspecies differences (log_2_(Cer/Par)) in modification levels for different regions along a gene (*upper panel*). Mean levels across three regions were taken: the promoter (‘*prom*’: −320 to −160 relative to the TSS), the respective loci with highest prevalence on average (‘*peak*’: 0 to +140 for H3K9ac, +100 to +580 for H3K4me3) and around the TTS (‘*end*’: −260 to +60 relative to the TTS), as indicated. The percentage of genes with absolute differences at these regions exceeding an increasing threshold (log_2_ scale) is depicted. **(D)** Comparison of intragenic differences in modification levels (log_2_(H3K9ac/H3K4me3)) between *S. cerevisiae* and *S. paradoxus* at orthologous genes. The interspecies correlation, and a linear fit of the data are shown. **(E)** Relative modification levels at orthologous genes for selected ontological groups. Shown are interspecies comparisons for ‘protein synthesis’ and ‘cytoskeleton’ genes (*upper panel*), and ‘ribosome’ and ‘RiBi’ genes (*lower panel*). **(F)** Scatter plots comparing ‘peak’ H3K9ac and ‘peak’ H3K4me3 between species. For each modification, the most divergent genes were extracted by applying the Lowess method to the interspecies plot, and selecting those genes farthest from the regression curve in either direction (c. 800 genes each; *upper left* and *lower right* panels). Genes with higher H3K9ac in *S. cerevisiae* (*dark green*) or *S. paradoxus (light green)* were overlaid onto the interspecies H3K4me3 plot (*upper right*), or vice versa (*lower left*). **(G)** Plot of interspecies differences in H3K9ac against H3K4me3. Genes showing consistent changes for both modifications (*black*), opposite changes (*grey*), no changes in either modification (*light grey*), changes in only in H3K9ac (*pink*), and changes only in H3K4me3 (*light blue*) are marked. Absolute differences above 0.4 (log_2_ scale) were considered as significant. The Pearson correlation and a linear fit for the data are shown. Relative proportions of each group are indicated in the adjacent pie chart.

Hence, we compared *S. cerevisiae* and *S. paradoxus*. Having diverged over 5my from a common ancestor, and exhibiting 80–90% sequence identity, these species are sufficiently close that the repertoire and chromosomal order of their genes is well preserved (shared synteny), yet sufficiently distant for widespread divergence of gene expression and its regulation [37], [38]. Genome-wide acetylation and methylation maps, as well as mRNA levels per gene, were generated for *S. paradoxus* as previously, and juxtaposed to those obtained for *S. cerevisiae*. Our analysis included c. 6000 pairs (90%) of genes, and sequencing reads were normalized for total read counts and log_2_ transformed. First, orthologous genes were compared by matching modification levels at corresponding loci (mean signal per 20 bp window); as shown, both H3K9ac and H3K4me3 were well correlated (r≈0.6 and r≈0.55, respectively; Figure 2A), indicating significant local agreement between species. Accordingly, average profiles for all genes were highly similar (Figure 2B, upper panels), supporting the notion that the characteristic genic distributions of H3K9ac and H3K4me3 are a recurrent design feature. To determine the degree of concordance across a gene, paired levels at each position were assessed for all genes. This revealed that the regions most enriched for acetylation (essentially +1nuc) and methylation (essentially +2nuc and +3nuc) were very well correlated between species (r≈0.6 and r≈0.7, respectively; Figure 2B, lower panel). Further, we detected remarkable agreement at the promoter particularly between H3K9ac levels (r≈0.7 and r≈0.5, respectively; Figure 2B, lower panel). However, given highly similar nucleosome occupancies between these species [24], significant correlation of promoter modifications may be an indirect consequence.

In the absence of well-defined consensus nucleosome positions for *S. paradoxus*, we considered three regions: the promoter (‘prom’: −320 to −160 relative to the TSS), the regions with highest abundance for each modification (‘peak’: 0 to +140 for H3K9ac, +100 to +580 for H3K4me3) and the end of genes (‘end’: −260 to +60 relative to the TTS). The extent of divergence at each region was then assessed; as shown, ‘peak’ acetylation and methylation (in particular) were best conserved, with only c.10% of genes displaying more than two-fold difference between the species despite high absolute levels within these regions (Figure 2C). H3K9ac differences at the end of genes were also restricted, likely due to lower signals in general, while promoter methylation was the most variable (almost 40% of genes with > 2-fold difference; Figure 2C). Hence, evolutionary conservation across a gene largely mirrors abundance and correlation with expression, reaffirming the functional significance of the respective patterns.

### Disparity of H3K9ac and H3K4me3 at orthologous genes is often maintained, yet global patterns of inter-species divergence between modifications appear largely independent

Given considerable interspecies agreement between individual modifications, we asked whether this level of conservation encompasses the intragenic disparity in *S. cerevisiae* (Figure 1). To address this, acetylation-to-methylation ratios were compared between species; indeed, they were well correlated (r≈0.5; Figure 2D), indicating that a significant proportion of genes retain disparity of modifications in evolution. We examined some of the strongest examples noted in *S. cerevisiae*; as shown, the prominent acetylation of protein synthesis genes and methylation of cytoskeletal genes are maintained, and a similar dichotomy survives for ‘ribosome’ vs ‘RiBi’ genes (Figure 2E). Overall, the marked evolutionary correlation for individual modifications is largely upheld in the context of their ratios, indicating the likely utility of asymmetric modifications. Predictably, similarly utilized orthologous genes are governed by similar regulatory constraints.

Apart from this basic association, however, the direct question of whether acetyl and methyl marks diverge together remains open. To approach this, we calculated a regression curve from the interspecies scatter plot for each modification, and extracted those genes farthest from the curve in either direction (Cer>Par and Par>Cer, c. 800 genes each). Coincidence of acetylation and methylation differences was then tested. As shown, overlap of genes showing both higher H3K9ac and H3K4me3 in the same species was only marginally significant (hypergeometric test, p≈0.03), and clearly, the overwhelming majority of genes (c. 85%) did not concur (Figure 2F). That is, divergence of one modification gave no indication for divergence of the other. We extended this analysis to all genes and plotted H3K9ac changes against H3K4me3 changes; as expected, there was only a nominal correlation between changes (Figure 2G; r≈0.1). Significant changes were then defined using a threshold of 1.3-fold (0.4 in log_2_ scale), encompassing c. 50% genes. Using this threshold, we noted that H3K9ac or H3K4me3 co-varied at only 17% of genes, while 22% showed no change in both modifications. Genes with differences only in acetylation were more prevalent than those only varying in methylation (27% and 21% of genes, respectively), and indeed, a significant fraction showed opposite changes (Figure 2G). Hence, transitions of intragenic H3K9ac and H3K4me3 levels between evolved states appear largely uncoordinated, with almost 50% of genes exhibiting variation in only one of the modifications. In this context, the retention of modification disparity at a fraction of genes may correspond to some level of mutual constraint, wherein acetylation levels vary within boundaries set by methylation levels, and *vice versa*. This may arise, for instance, by selective engagement of cross-talk mechanisms. However, the overwhelming lack of correlation indicates that acetyl and methyl levels are governed by largely independent sets of genetic determinants, which in turn allow considerable functional autonomy.

### Changes in H3K4me3 coincide with expression divergence more often than do H3K9ac changes

**Figure 3:**
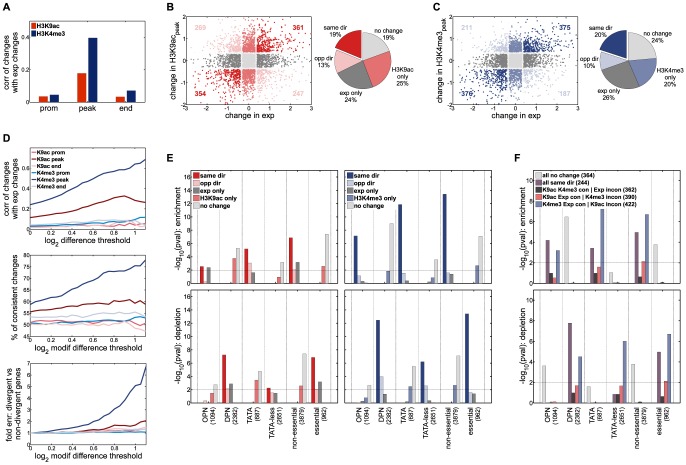
Interspecies differences in H3K4me3, compared to H3K9ac, better predict expression divergence. **(A)** Correlation between interspecies expression differences and either H3K9ac (*red*) or H3K4me3 (*blue*) changes, at different regions along a gene, as indicated. Correlations were calculated for significant changes in expression and modifications (absolute differences greater than 0.4 (log_2_ scale)). **(B)** Scatter plot of interspecies differences in mRNA levels against changes in ‘peak’ H3K9ac. Genes showing consistent changes between *S. cerevisiae* and *S. paradoxus* (*dark red*), opposite changes (*pink*), no changes in either parameter (*light grey*), changes in only in H3K9ac (*light red*), and changes only in expression (*dark grey*) are marked. The numbers of genes showing consistent or opposite changes are noted, and the relative proportions of each group are indicated in the adjacent pie chart. **(C)** As in (B), but for H3K4me3 changes. Genes showing consistent changes (*dark blue*), opposite changes (*light blue*), no changes in either parameter (*light grey*), changes in only in H3K9ac (*blue*), and changes only in expression (*dark grey*) are marked. **(D)** *Upper panel*, Interspecies correlation between expression differences and H3K9ac (*red*) or H3K4me3 (*blue*) differences at different regions along a gene, as indicated, calculated at increasing absolute thresholds (for both expression and modification changes). *Middle panel*, Graph depicting the proportion of genes for which interspecies changes in expression and modifications (at different regions) are consistent (in the same direction). Percentages were calculated for an increasing threshold of absolute modification differences, and considering significant expression differences (> 0.4). *Lower panel,* Genes with the largest and smallest differences in expression *S. cerevisiae* and *S. paradoxus* (‘*divergent*’ and ‘*non-divergent*’; 1000 genes each) were taken. Thereafter, for an increasing threshold of absolute differences in modifications at different regions, the fold enrichment of divergent over non-divergent genes was calculated at each threshold. **(E)** Enrichment (*upper panels*) or depletion (*lower panels*) of sets of genes defined according to patterns of H3K9ac/mRNA changes (*left*) or H3K4me3/mRNA changes (*right*) amongst the indicated architectural or phenomenological gene classes; *p* values were calculated using a hypergeometric test. **(F)** Genes were classified based on interspecies variation in all three parameters (H3K9ac, H3K4me3, expression): those showing no change in either (*light grey*), consistent changes amongst all three (*purple*), consistent changes between H3K9ac and H3K4me3 but not expression (*dark grey*), consistent changes between H3K9ac and expression but not H3K4me3 (*light red*), and those showing consistent changes between H3K4me3 and expression but not H3K9ac (*blue*). Gene sets were subsequently analyzed as in (E).

In light of their dissimilar evolutionary profiles, the question as to which histone mark diverges more closely with expression is raised; here, genes with significant changes between *S. cerevisiae* and *S. paradoxus* in both mRNA levels and modifications were considered (at least 1.3-fold). As shown, expression differences correlated significantly better with changes in ‘peak’ H3K4me3, as compared to ‘peak’ H3K9ac (r≈0.4 and r≈<0.2, respectively; Figure 3A). Examining this further, all genes were classified according to whether they vary in expression, modifications (average signal for the ‘peak’ regions) or both. The proportion of genes that co-varied with expression was similar for both modifications (c.20%), but H3K9ac, relative to H3K4me3, showed a higher fraction of changes not coordinated with expression (modification changes only or opposite changes: 38% vs 30%; Figure 3B and 3C).

On further analysis, the better predictive value of ‘peak’ methylation over ‘peak’ acetylation is clarified. First, correlation with methylation differences consistently rises with an increasing threshold for absolute changes (e.g. r≈0.6 for genes with greater than two-fold difference), but association with acetylation plateaus at significantly lower correlations (maximally, r≈0.3; Figure 3D, top panel). Accordingly, for genes with significant expression differences (at least 1.3-fold), the percentage of genes with consistent H3K4me3 changes (that is, in the same direction) outweighs those for H3K9ac at all taken thresholds (e.g. 75% compared to <60% with consistent changes, respectively, for a two-fold threshold; Figure 3D, middle panel). Further, we selected the most and least divergent genes with respect to expression (1000 genes each), and compared their presence for a range of modification differences. Clearly, the fraction of divergent genes far outweighed that of non-divergent genes for methylation, but not for acetylation, across the scale of difference thresholds tested (Figure 3D, bottom panel); for instance, a two-fold threshold for H3K4me3 differences incorporated a 4.5-fold enrichment in the fraction of divergent over non-divergent genes, while H3K9ac coupled to only a 1.5-fold enrichment. Hence, between closely related yeasts, methylation downstream of the TSS often varies together with mRNA levels, but association with TSS acetylation changes is significantly weaker.

### Particular gene attributes favor coordination of H3K4me3 and expression divergence

Interspecies variation in methylation, in particular, may disclose the degree of expression divergence, but this relationship is only upheld for a fraction of the gene pool (c.40%). Hence, we asked whether certain gene attributes might be favored amongst these genes, and tested association with nucleosome organization (OPN vs DPN) and promoter sequence (TATA-containing vs TATA-less). Amongst genes for which mRNA and H3K4me3 changes were consistent (in the same direction between species), both TATA-containing and OPN genes were highly enriched (p≈10^−12^ and p≈10^−7^, respectively), while DPN and TATA-less genes were significantly depleted (p≈10^−12^ and p≈10^−6^, respectively; Figure 3E). As there is considerable overlap between OPN and TATA-containing genes, we looked at intersecting gene groups to determine the dominant feature; as depicted, evolutionary coordination of expression with methylation is favored when both features are present (Figure S6 in File SI). This is to be expected given that OPN and TATA-containing genes are often divergently expressed in evolution. However, it is notable that these genes were not significant when expression changes were not accompanied by consistent H3K4me3 changes (Figure 3E, and Figure S6 in File SI). Furthermore, H3K9ac marks did not recapitulate these biases; for instance, TATA-containing genes showed both consistent and opposite changes with regards to expression divergence (p≈10^−5^ and p≈10^−3^; respectively), and OPN genes were equally present whether or not H3K9ac levels co-varied with expression (p≈10^−2^; Figure 3E).

Essential and non-essential genes were similarly analyzed, and based on their evolutionary proximity, we assumed equivalent gene cohorts in *S. cerevisiae* and *S. paradoxus*. Similar to OPN and TATA-containing genes, non-essential genes were highly prevalent when methylation and mRNA levels varied together (p≈10^−13^), but not amongst genes with uncoordinated differences. For H3K9ac, non-essential genes also distributed amongst those with inconsistent changes. Differences between the modifications are even more pronounced when assessing H3K9ac, H3K4me3 and mRNA changes concurrently. For instance, TATA-containing genes appear enriched amongst those that exhibit consistent methylation and expression changes between species, regardless of whether or not acetylation changes are in agreement, but not vice versa (Figure 3F). In sum, variation in methylation, but not acetylation, appears to be well coordinated with expression divergence at TATA-containing, OPN and non-essential genes. At such genes, evolution of expression might have entailed modulation of H3K4me3 levels.

For a functional perspective, genes grouped by gene ontology or ‘transcriptional modules’ (as described earlier) were also examined. This revealed that gene sets with coordinated changes are largely common to both acetylation and methylation. For instance, higher modification and expression in *S. cerevisiae* was significant for amino acid biosynthesis genes; for *S. paradoxus*, higher modification/expression levels were prevalent at mitochondrial genes, in line with the differential respiratory strategies of the species under rich conditions (Figure S7 in File SI). In contrast, several sets were inconsistent with respect to acetylation, but not methylation. For instance, genes engaged in protein synthesis tended to be differentially expressed without variation in H3K9ac levels; ribosomal RNA-associated genes, which were invariantly expressed, were also inclined towards invariant H3K4me3, but not invariant H3K9ac. Other gene sets (e.g. bud-neck, nuclear, nucleolar and cell cycle genes) also tended towards variable acetylation with no change in expression (Figure S7 in File SI). Hence, examination of functionally coordinated groups also highlights a propensity for incoherent acetylation and expression differences, whereas methylation tends to concur for a greater number. Overall, these results raise the notion that, at divergently expressed orthologous genes, H3K4me3 may have been enlisted as a means to stabilize interspecies differences in mRNA levels. Poorer evolutionary coordination with H3K9ac, on the other hand, indicates indirect involvement, conceivably, in the temporal regulation of transcription.

## Discussion

**Figure 4:**
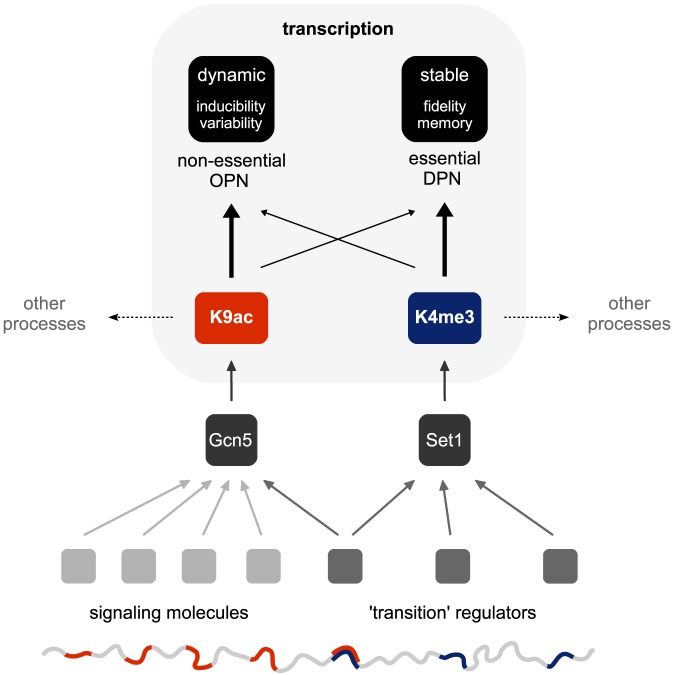
Selective utilization and evolutionary divergence of transcription-linked histone modifications. Scheme depicting the differential distribution of H3K9ac and H3K4me3 marks amongst the gene pool in budding yeast. Higher acetylation manifests at non-essential OPN genes, while methylation appears enriched at essential DPN genes. We propose that these patterns reflect disparate blueprints of transcriptional regulation. That is, selective engagement of acetylation at the TSS may enable a dynamic program, varying both temporally and quantitatively, through control of transcription induction. H3K4me3 may engender higher fidelity and consistency of transcription, and so contribute to a more stable program. This division of labor is corroborated in comparisons between related yeast species. In particular, H3K9ac and H3K4me3 marks appear to vary independently of each other, yet interspecies changes in steady-state expression are well explained by differences in methylation, rather than acetylation. Hence, evolutionary regulation of these marks likely involves genetic changes at largely non-overlapping sets of *trans* factors. Conceivably, mutations at a variety of signaling molecules that react to environmental perturbations could impinge on Gcn5 acetylase activity, whereas modulation of Set1 may involve changes at a more select set of regulators engaged in large-scale phenotypic transitions (such as cell division or differentiation).

Histone acetylation and methylation are central players in the transcriptional process, yet as distinct chemical moieties, each is likely to embody specialized functions. In this study, we compared the distributions of H3K9ac and H3K4me3 marks at high resolution in budding yeasts, in order to discover distinguishing features in the context of gene expression. Differences were manifested at several levels. First, each mark exhibited characteristic gene patterns: acetylation concentrated at TSS nucleosomes, and methylation, at proximal ORF nucleosomes. Second, we found that these marks were asymmetrically distributed amongst the gene pool: H3K9ac appeared more prominently at non-essential, responsive genes and an OPN architecture, while higher H3K4me3 was enriched at essential, periodically expressed and DPN genes (Figure 4). Third, both acetylation and methylation patterns were well conserved between *S. cerevisiae* and *S. paradoxus*, but their divergence at orthologous genes appeared largely uncoordinated with each other, indicating largely independent evolutionary trajectories of their respective machineries. Finally, expression divergence, particularly at OPN and TATA-containing genes, was well explained by changes in H3K4me3, but not H3K9ac, suggesting methylation as a plausible evolutionary candidate to tune transcriptional output.

In our initial survey of gene ontology/transcriptional modules, it was interesting to note that higher methylation, relative to acetylation, appeared to manifest preferentially at gene cohorts with ‘housekeeping’ or structural roles, such as the biogenesis and/or maintenance of organelles and the cytoskeleton (Figure 1D and 1E, and Figure S1F in File SI). A similar preference was also found when classifying genes in terms of essentiality and periodicity between cell cycles (Figure 1G). Therefore, we considered that higher aggregate H3K4me3 levels might relate to the need to ensure that such genes are to be infallibly and consistently expressed between successive cell generations. Indeed, a growing body of work from across the eukaryotic divide associates the H3K4me3 mark with stable transcription. Using live imaging to follow transcription events at a single housekeeping gene in individual *Dictyostelium* cells, Muramoto et al. showed that frequency of transcriptional pulses tended to be inherited between successive cell generations, and that H3K4me3 marks were important in maintaining the memory of active states [39]. In a similar vein, diverse studies attribute to H3K4me3 a role in preserving and/or reflecting the characteristic expression profiles of differentiated cells. These include key involvement in persistent expression of somatic genes in nuclear transplantation experiments [40], and maintaining homeostasis in adult cardiomyocytes [41]. Interestingly, a recent report, charting the proximity of various histone modifications with single nucleotide polymorphisms (SNPs) linked to complex human traits, found H3K4me3 to be the most informative for identifying the participant cell types [42], again, coupling this mark to the phenotype of specialized cells.

In line with conferring memory of expression, the dynamics of histone methylation are believed to be markedly slower than those of acetylation [43]; turnover rates for acetylation are typically in the order of minutes [44]. H3K4me3 marks, on the other hand, are likely more dependent on DNA replication for their erasure, and may linger for hours beyond the residence time of an actively transcribing Pol II [45]–[47]. Further, persistent methylation has been found to obstruct reactivation of recently transcribed genes [48], thereby curbing transcriptional output. Indeed, increasing evidence attributes to H3K4me3 repressive roles in gene expression, through several mechanisms. For instance, elevated gene expression upon deletion of the methylase, Set1, has been recently linked to de-repression of attendant antisense transcription [49]–[51]. Further, methylation marks, including H3K4me2/3 and H3K36me2/3, are thought to decrease accessibility of the local chromatin structure, often via direct association with various HDACs. Chromatin compaction may serve various ends, including reducing the processivity of an elongating Pol II, protecting against cryptic initiation of transcription, and preventing basal gene activation [15]. Plausibly, such buffering mechanisms converge on a common evolutionary objective – to impart fidelity, both in terms of Pol II accuracy and in delimiting mRNA output boundaries.

If H3K4me3 marks contribute to constrained but reliable gene transcription, then the synchrony with expression changes that we observed between related yeasts (Figure 3) may be readily explained. Conceivably, evolution has enlisted H3K4me3 as a means to stabilize gene output at levels suitable to the adapted species: that is, robust interspecies transitions in the expression of orthologous genes may have been partly achieved by modulating histone methylation. It is interesting to note that other features of the transcriptional process have not been found to concur evolutionarily. For instance, sequence divergence at TF binding sites explains only a small fraction of interspecies expression differences [25]. Likewise, although nucleosome positioning at gene promoters often correlates with expression changes between different environments and cell types [5], [52], [53], this relationship is not upheld in evolutionary timescales [24]. Namely, the genetic basis of expression divergence does not appear to encompass regulation of nucleosome positioning, but may rather involve mutations that impinge on the H3K4me3 marks, amongst other features to be identified. Hence, this class of chromatin features may serve as a rheostat for appropriate gene yield, whilst overriding discrepancies of other features such as nucleosome positioning and TF binding sites.

Acetylation presents a different picture in our analyses. Unlike H3K4me3, H3K9ac marks concentrated at TSS nucleosomes (Figure 1A), which co-localize with the transcription initiation machinery. Moreover, H3K9ac was generally prevalent at non-essential genes, as well as at responsive genes intrinsically capable of variable expression in different environments (Figure 1G). Protein synthesis and ribosomal protein (RP) genes, which are highly expressed in response to nutrient signals in exponentially growing cells, were also targets for hyper-acetylation (Figure 1E, and Figure S1F in File SI). Further clues into the utility of excess acetylation emerge from considering OPN/DPN status. In general, this classification of genes has proved insightful in gene regulation: OPN genes tend to incorporate greater regulatory complexity, including gene-tailored TFs, and presumably, promoter nucleosome-remodeling activities, which, in turn, enables inducible, conditional gene expression [27], [36]. The enrichment of H3K9ac over H3K4me3 at OPN-structured genes, and its relative dearth at DPN genes (Figure 1F), may therefore be a consequence of selective recruitment of acetylation enzymes. Indeed, although likely integral to general transcription, the HAT-bearing SAGA complex is often specifically engaged by multiple transcription factors in response to inductive signaling [54]. Further, several components of SAGA may substitute for components of the general transcription machinery (TFIID), particularly during transcription of stress-induced genes [55]. Hence, where H3K4me3 marks are perhaps more generically employed and less amenable to change, acetylation machineries appear highly sensitive to signaling, linking H3K9ac (and other histone acetyl marks) with timely induction of transcription [34].

Acetylation dynamics that are far above the rate of histone turnover, by definition, implies the continuous engagement of both HAT and HDAC activities. Interestingly, this reversibility is thought to be important to leverage responsiveness. For instance, deletion of a component of the RPD3L deacetylase complex, which is recruited to gene promoters, impaired both induction and repression of ESR genes in response to stress [56]. Early studies showed that the coding region deacetylase complex, SET3, was required for efficient activation of the GAL gene cluster [57]. Hence, dynamic acetylation at activated genes may serve to reset chromatin template between successive rounds of transcription, thereby creating conditions for efficient re-initiation.

In this light, the poorer association we found between divergence of H3K9ac marks and expression in related yeasts (Figure 3) may be rationalized. If acetylation impinges primarily on the efficiency/kinetics of gene induction, then it is reasonable to expect that its links to steady-state expression dissipate during evolution. For example, several groups of genes presented differences in H3K9ac without varying in expression (Figure S7 in File SI); such genes may be encountering different efficiencies of transcription, according to given conditions in the given species, but the cumulative outcome on expression levels may ultimately be unchanged. Accordingly, when inspecting simultaneous modifications, the presence of H3K9ac marks did not improve the predictive power of H3K4me3 changes in expression divergence (Figure 3F). Genes pertaining to oxidative phosphorylation were a clear exception in this regard (Figure S7 in File SI), but given that the preferred respiratory programs of *S. cerevisiae* and *S. paradoxus* (anaerobic vs aerobic respiration, respectively) are most likely hard-wired through signaling, interspecies differences in the efficiency of gene induction, in addition to output, might be expected. Our hypothesis concurs well with a study examining natural variation in H3K14 acetylation between different *S. cerevisiae* strains; inter-strain differences were also not indicative of expression divergence, but H3K14ac changes were enriched at responsive genes [58].

It is interesting to speculate on the evolutionary aspects of our findings. At orthologous genes, H3K9ac changes were only nominally correlated with H3K4me3 changes (r≈0.1; Figure 2F and 2G). This implies that interspecies genetic differences that impinge on each mark are largely non-overlapping, and have probably evolved independently. On the other hand, higher coordination of H3K4me3 and steady-state mRNA changes, especially at TATA-containing and OPN genes (Figure 3A and 3E), indicates that the methylation and transcription machineries are to some extent affected by a common pool of mutations. What then is the nature of such mutations? Given general pervasiveness of chromatin features, and their specific utility as modulators of gene expression, it seems unlikely that their genetic regulation involves many individual changes in the proximity of target genes (*cis* effects). Rather, mutations that affect the activity/expression of regulatory factors (*trans* effects) are more plausible evolutionarily. Previous work employing an interspecific hybrid strain to distinguish *cis*/*trans* contributions to expression divergence strongly corroborates this assumption: although *cis* effects were generally more prevalent, *trans* regulation predominated at genes most affected by deletion of chromatin regulators [38]. Likewise, expression evolution of intrinsically divergent OPN and TATA-containing genes was also better explained by *trans* rather than *cis* effects [23], [38]. Obvious candidates for evolutionary selection are core components of the H3K4 methylation complex (COMPASS). However, other factors that exert leverage over this machinery are also likely to have been enlisted. The recurrent association of H3K4me3 marks with steady-state phenotype, together with their relative stability, perhaps predicts that potential evolutionary targets will have been limited to a select type; namely, those capable of driving large-scale cellular transitions, such as division or differentiation (Figure 4).

What might be the genetic basis of H3K9ac marks? As with other chromatin regulators, *trans* effects that originate at the enzymatic machinery are likely. However, compared to methylation, a variety of ancillary factors might be expected. Rapid dynamics, together with a posited role in transcription induction, predicts that acetyl marks are sensitive to multiple signaling pathways, including those that mediate prompt adaptation to environmental perturbations. Accordingly, a large collection of mutations at upstream signaling molecules may have contributed to H3K9ac divergence (Figure 4), which is in line with a weaker association with expression divergence (Figure 3). Furthermore, H3 acetylation and H3K4 methylation are engaged in other DNA-centered processes, including replication and recombination [59], [60], indicating additional complexity into the genetic regulation of these marks in the context of transcription.

## Conclusions

Overall, our results show that distinct histone modifications appear to be deployed selectively across the gene pool, in a manner that associates with expression variability on the one hand (higher H3K9ac at the TSS) and stability on the other (higher H3K4me3 at proximal ORF nucleosomes). Our comparative inter-species analysis corroborates their independent regulation, and proposes H3K4me3, rather than acetylation, as a possible evolutionary means to control expression divergence. In future work, it will be interesting to test *cis* and *trans* contributions to expression regulation by profiling an interspecific hybrid. Moreover, current high-throughput ChIP-seq technologies make it feasible to extend this comparative approach to multiple chromatin features in parallel, including other histone marks, nucleosome remodelers and histone chaperones. This will help to unravel the interfaces that link mutations, target molecules, functional pleitropy, and cellular phenotype.

## Supporting Information

File S1 **Figure S1**, Genome-wide features of H3K9ac and H3K4me3 marks in *S. cerevisiae*. **Figure S2**, H3-normalization of H3K9ac and H3K4me3 levels in S. cerevisiae. **Figure S3**, Disparity of H3-normalized H3K9ac or H3K4me3 levels per gene: analysis of expression-correlated regions. **Figure S4**, Disparity of H3-normalized H3K9ac or H3K4me3 levels per gene: analysis across the proximal ORF. **Figure S5**, Analysis of H3K9ac/H3K4me3 disparity amongst various gene subsets. **Figure S6**, Association of gene architecture with evolutionary coordination between histone modifications and expression. **Figure S7**, Co-divergence of histone modifications with expression in the context of gene ontology.
(PDF)
