# Supplementary material for: Divergence and Selectivity of Expression-Coupled Histone Modifications in Budding Yeasts
Source: PLoS One. 2014 Jul 9;9(7):e101538. doi: 10.1371/journal.pone.0101538 (PMC4090005; doi:10.1371/journal.pone.0101538)
Supplement: File S1 — Figure S1, Genome-wide features of H3K9ac and H3K4me3 marks in S. cerevisiae. Figure S2, H3-normalization of H3K9ac and H3K4me3 levels in S. cerevisiae. Figure S3, Disparity of H3-normalized H3K9ac or H3K4me3 levels per gene: analysis of expression-correlated regions. Figure S4, Disparity of H3-normalized H3K9ac or H3K4me3 levels per gene: analysis across the proximal ORF. Figure S5, Analysis of H3K9ac/H3K4me3 disparity amongst various gene subsets. Figure S6, Association of gene architecture with evolutionary coordination between histone modifications and expression. Figure S7, Co-divergence of histone modifications with expression in the context of gene ontology. (PDF) [file pone.0101538.s001.pdf]

Supplementary figures

Figure S1. **Genome-wide features of H3K9ac and H3K4me3 marks in *S. cerevisiae*. (A)** Distribution of average H3K9ac and H3K4me3 levels at the gene promoter (*'prom'*, *pink* and *light blue*, respectively) and across the open reading frame (*'ORF'*, *red* and *blue*, respectively) for all genes. **(B)** Distribution of H3K9ac (*left panel*) and H3K4me3 (*right panel*) levels at nucleosome positions along a gene. Shown is the cumulative fraction of genes with modification levels at the selected nucleosomes ('*-1*', '*+1*', '*+2*', '*+3*', '*+4*', '*+5*', as indicated) less than a given increasing threshold. **(C)** Trends of H3K9ac (*left panel*) and H3K4me3 (*right panel*) levels at selected nucleosomes, as indicated, sorted by increasing nucleosome occupancy. Mean modification values within a moving window of 500 genes were plotted. **(D)** Graphs showing the mean H3K9ac (*left panel*) and H3K4me3 (*right panel*) profiles around the TSS for genes classed according to their level of expression in rich media. The legend indicates the median mRNA expression ($\log_2$ scale) in each group, as determined by RNA sequencing. **(E)** *Left panel*, Plot of H3K9ac (at +1nuc) against mean H3K4me3 (across +2nuc and +3nuc). The calculated Pearson correlation between the modifications and linear fit of the data are shown. *Right panel*, as in *left panel*, but after normalizing the data to expression levels. For normalization, local regression was applied to the respective modification-vs-expression graphs using the Lowess method, and modification values then subtracted from the fitted curve. **(F)** Examples of ontological classes of genes biased towards higher H3K9ac relative to H3K4me3, and *vice versa*. All genes were ranked according to the ratio of genic H3K9ac/H3K4me3 levels (*light grey curve*). The placing along the curve of individual genes belonging to the indicated ontology class is marked (*grey circles*).

Figure S2. **H3-normalization of H3K9ac and H3K4me3 levels in S. cerevisiae. (A)** Examples of H3K9ac (*red*) and H3K4me3 (*blue*) profiles (at genes, HYS2 and PPG1) prior to (*upper panels*) and following (*lower panels*) normalization by H3 levels (*middle panels*). Normalization was carried out by subtracting $\log_2$(H3) from $\log_2$(modification) levels at each binned position (mean signal across 20bp intervals). **(B)** H3K9ac and H3K4me3 gene profiles around the TSS and TTS averaged across all genes before (*upper panel*) and after (*lower panel*) H3 normalization. The middle panel shows the average H3 profile for all genes. **(C)** Graphs showing the mean, H3-normalized H3K9ac (*upper panel*) and H3K4me3 (*middle panel*) profiles around the TSS for genes classed according to their level of

expression in rich media. The legend indicates the median mRNA expression (log_2 scale) in each group, as determined by RNA sequencing. *Bottom panel*, correlation of H3-normalized H3K9ac and H3K4me3 with expression along a gene. For all genes, the mean H3K9ac and H3K4me3 level was taken in a moving window (of 140 bases), normalized by the corresponding average H3 signal (log_2(modification/H3)), and the Pearson correlation per window calculated and plotted.

Figure S3. **Disparity of H3-normalized H3K9ac or H3K4me3 levels per gene: analysis of expression-correlated regions.**

(A) Scheme depicting the gene regions examined for differential modification levels after normalization by H3. Average H3K9ac and H3K4me3 across the indicated regions (-60 to +140, and +100 to +580 relative to the TSS, respectively), were normalized by the corresponding average H3 levels (log_2(modification/H3)), and then analysed for enrichment of various gene features, as below. **(B)** The ratio between H3-normalized H3K9ac and H3K4me3 was calculated, and genes were then ranked accordingly and plotted (*left panel*); three sectors (1200 genes each) were then considered: H3K4me3 > H3K9ac (*blue*), H3K4me3 ≈ H3K9ac (*grey*), and H3K4me3 < H3K9ac (*red*). Enrichment of various categories of genes ('GO slim' categories, www.geneontology.org) or by pre-defined transcriptional modules [30]) within each of these sectors was then assessed using a hypergeometric test. Significantly enriched categories ($-1*log_{10}(pval) > 2$) are depicted in the bar graphs. **(C)** Genes were classified according to their promoter nucleosome architecture (occupied proximal nucleosome, *'OPN'*; depleted proximal nucleosome, *'DPN'*), or according to whether or not they incorporate a TATA-box within the promoter (TATA-containing, *'TATA'*; or TATA-deficient, *'Tless'*). Thereafter, enrichment (*upper left panel*) or depletion (*upper middle panel*) of these classes amongst the sectors defined in terms of the genic H3-normalized H3K9ac/H3K4me3 ratio (as in (B)) was calculated using a hypergeometric test (*top panel)*. Calculated p values are shown as -1*log10(pval)). The lower left panel depicts enrichment/depletion as a percentage of the expected abundance (according to the null hypothesis); error bars indicate the standard deviation from the expected mean incidence. *Right panels*, and in left panels, but assessing the enrichment/depletion of genes classified according to several features of expression: '*responsive*' and '*non-responsive*' genes, defined by their expression variance across a large compendium of conditions [30]; '*periodic*' genes, which show cyclical expression between

successive cell cycles (800 genes; as defined in [29]); '*essential*' and '*non-essential*' genes (defined according to the viability in rich media of their respective deletion mutants).

Figure S4. **Disparity of H3-normalized H3K9ac or H3K4me3 levels per gene: analysis across the proximal ORF.**

(A) Scheme depicting the gene region examined (-60 to +580 relative to the TSS) for differential modification levels after normalization by H3. Average H3K9ac and H3K4me3 levels across the indicated region were normalized by the corresponding average H3 levels ($\log_2$(modification/H3)), and then analysed for enrichment of various gene features, as below. **(B)** As in Figure S3B. **(C)** As in Figure S3C.

Figure S5. **Analysis of H3K9ac/H3K4me3 disparity amongst various gene subsets. (A)** Gene sets (1200 genes each) exhibiting higher relative H3K9ac levels (*red*), higher relative H3K4me3 (*blue*), or equivalent H3K9ac/H3K4me3 levels (*grey*), were assessed for enrichment or depletion of subsets of OPN (*left*) or DPN (*right*) genes further classified according to various other characteristics (*'TATA'*, *'Tless'*, *'responsive'*, *'periodic'*, *'non-essential'*, *'essential'*). Enrichment (*upper panels*) or was determined using a hypergeometric test (p values shown as -1*log10(pval)). The number of genes in each subgroup is indicated. The lower panel shows enrichment/depletion as a percentage of the expected abundance (according to the null hypothesis); error bars indicate the standard deviation from the expected mean incidence. **(B)** Enrichment (*upper panels*) or depletion (*lower panels*) as in (a), but for selected ontological classes further subdivided according to OPN/DPN status, as indicated.

Figure S6. **Association of gene architecture with evolutionary coordination between histone modifications and expression.** The tendency of combinations of gene features (OPN/DPN architecture together with the presence of absence of a TATA-box within the promoter) to associate with coordinated divergence of H3K9ac or H3K4me3 marks with expression was assessed. Enrichment (*upper panel*) or depletion (*middle panel*) of these features amongst genes exhibiting consistent or inconsistent changes (as described in Figure 3b and 3c) was examined as previously. The lower panel shows enrichment/depletion as a percentage of the expected abundance; error bars indicate the standard deviation from the

expected mean incidence.

Figure S7. **Co-divergence of histone modifications with expression in the context of gene ontology.** Enrichment of gene ontology classes amongst different H3K9ac/mRNA (*left panels*) and H3K4me3/mRNA (*right panels*) divergence profiles, as indicated; namely, genes with consistent changes in *S. cerevisiae* (*'sdir (Cer)'*), genes with consistent changes in *S. paradoxus* (*'sdir (Par)'*), genes with changes in expression only ('*exp only*'), genes for which only modifications change ('*H3K9ac only*' or '*H3K4me3 only*'), and genes with no change in either expression or modification ('*no change*'). Gene classes significantly enriched in each profile (hypergeometric test, $-\log_{10}$(pval) >2) are depicted.

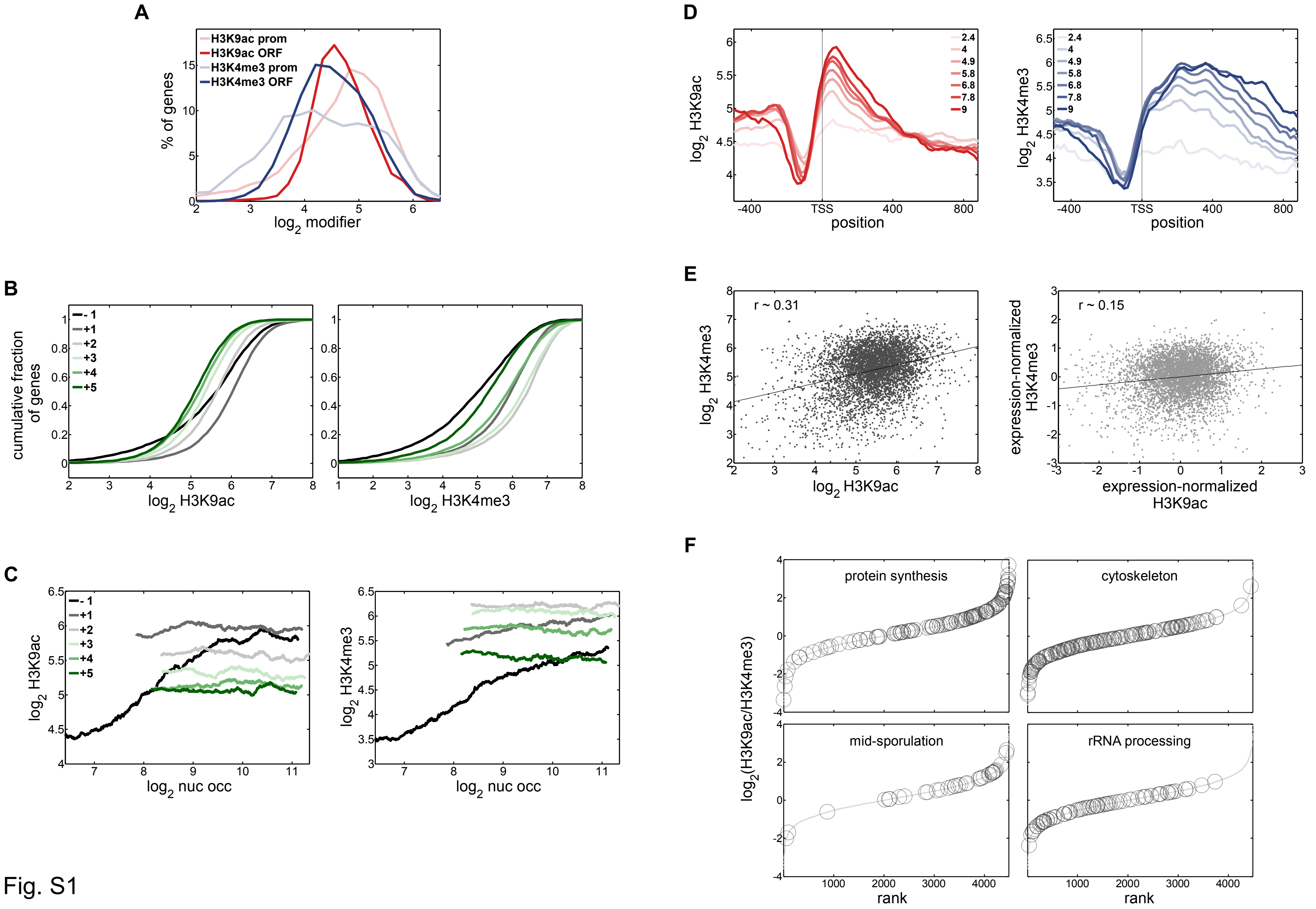

A
H3K9ac prom
H3K9ac ORF
H3K4me3 prom
H3K4me3 ORF
% of genes
log2 modifier
B
cumulative fraction of genes
log2 H3K9ac
log2 H3K4me3
C
log2 H3K9ac
log2 H3K4me3
log2 nuc occ
D
log2 H3K9ac
log2 H3K4me3
position
TSS
E
r ~ 0.31
r ~ 0.15
log2 H3K4me3
log2 H3K9ac
expression-normalized H3K4me3
expression-normalized H3K9ac
F
protein synthesis
cytoskeleton
mid-sporulation
rRNA processing
log2(H3K9ac/H3K4me3)
rank

Fig. S1

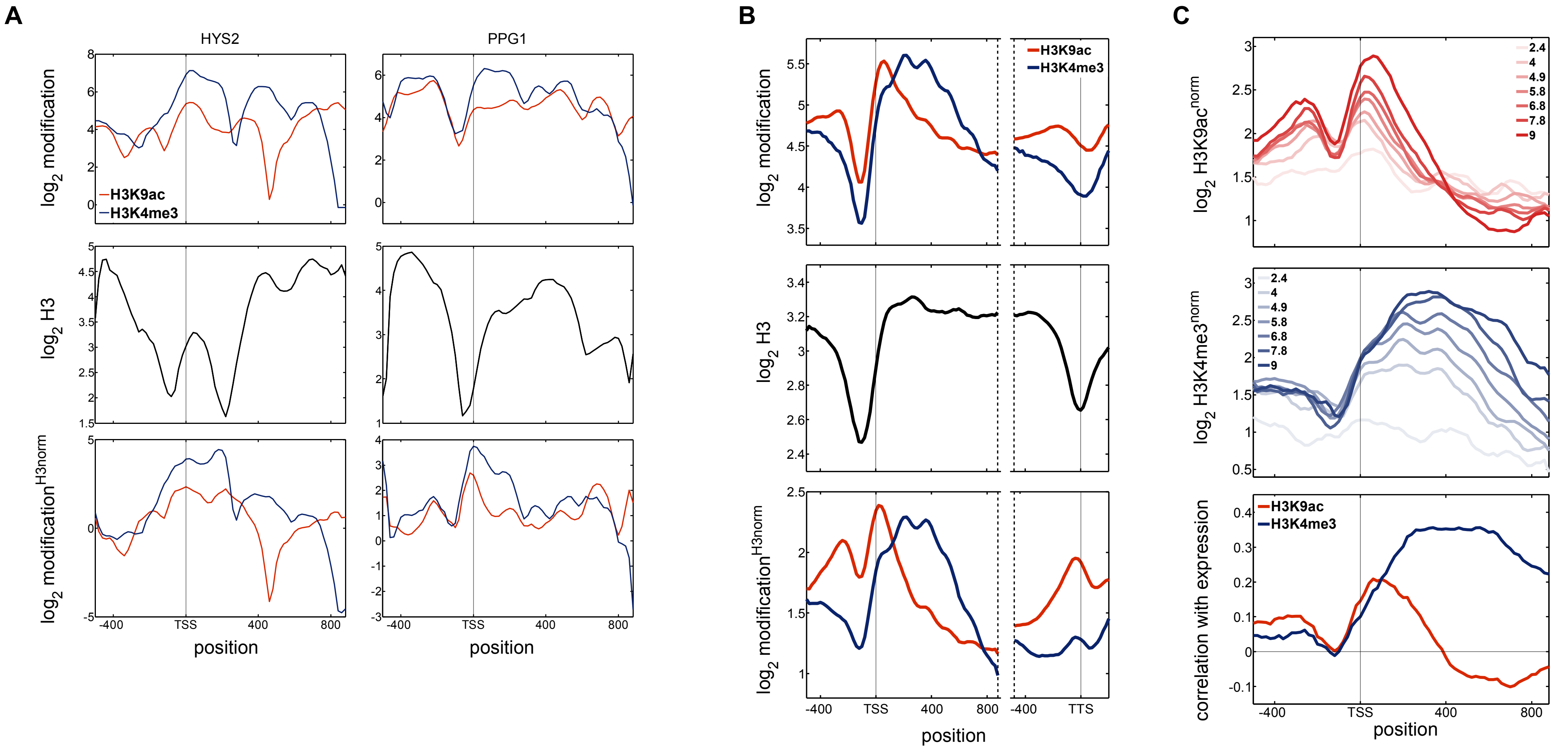

Fig. S2

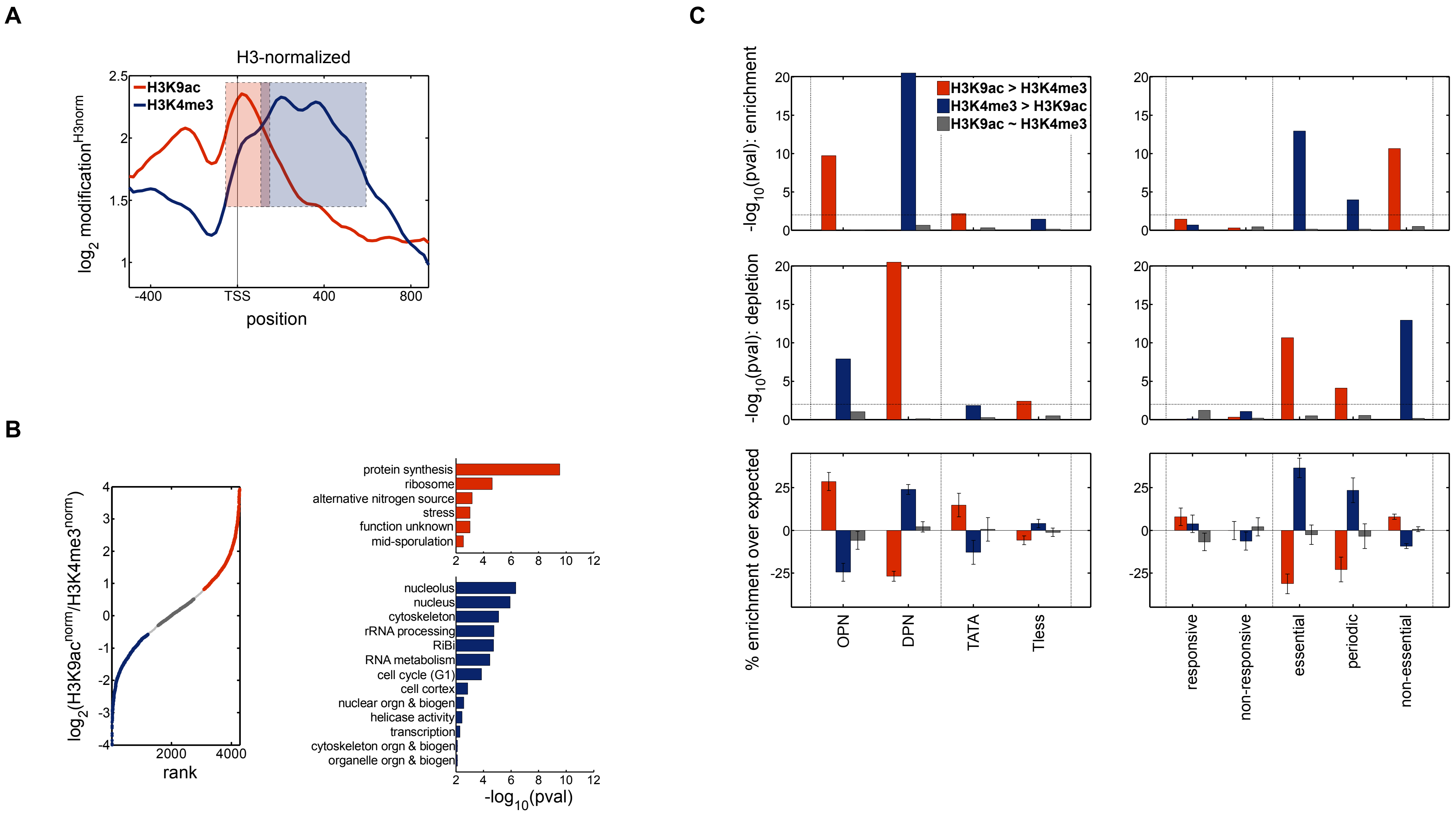
A
H3-normalized
H3K9ac
H3K4me3
log2 modification H3norm
TSS
position
B
log2(H3K9ac norm/H3K4me3 norm)
rank
protein synthesis
ribosome
alternative nitrogen source
stress
function unknown
mid-sporulation
nucleolus
nucleus
cytoskeleton
rRNA processing
RiBi
RNA metabolism
cell cycle (G1)
cell cortex
nuclear orgn & biogen
helicase activity
transcription
cytoskeleton orgn & biogen
organelle orgn & biogen
-log10(pval)
C
H3K9ac > H3K4me3
H3K4me3 > H3K9ac
H3K9ac ~ H3K4me3
-log10(pval): enrichment
-log10(pval): depletion
% enrichment over expected
OPN
DPN
TATA
Tless
responsive
non-responsive
essential
periodic
non-essential

Fig. S3

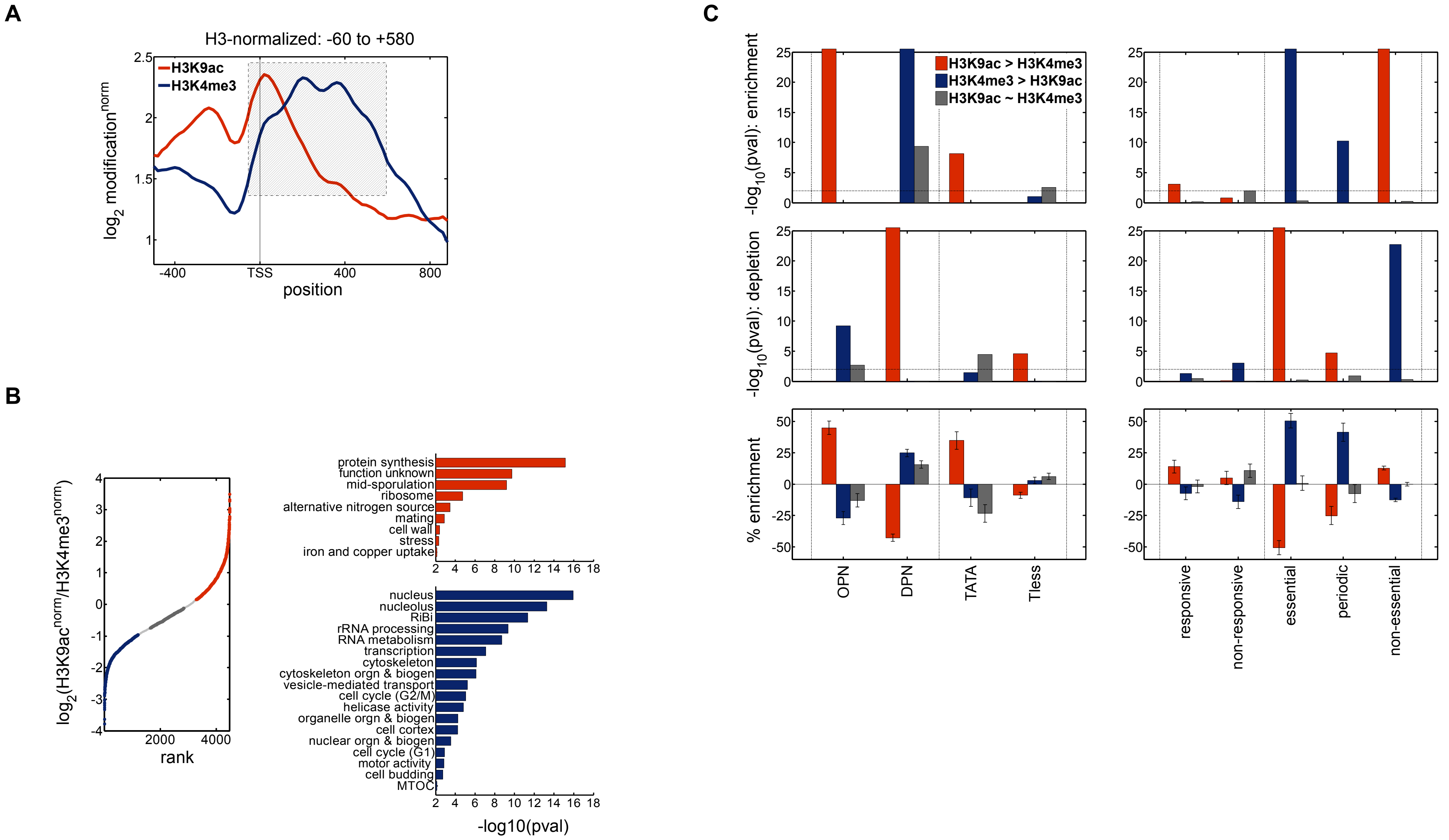
A
H3-normalized: -60 to +580
H3K9ac
H3K4me3
log2 modification^norm
position
B
log2(H3K9ac^norm/H3K4me3^norm)
rank
protein synthesis
function unknown
mid-sporulation
ribosome
alternative nitrogen source
mating
cell wall
stress
iron and copper uptake
nucleus
nucleolus
RiBi
rRNA processing
RNA metabolism
transcription
cytoskeleton
cytoskeleton orgn & biogen
vesicle-mediated transport
cell cycle (G2/M)
helicase activity
organelle orgn & biogen
cell cortex
nuclear orgn & biogen
cell cycle (G1)
motor activity
cell budding
MTOC
-log10(pval)
C
H3K9ac > H3K4me3
H3K4me3 > H3K9ac
H3K9ac ~ H3K4me3
-log10(pval): enrichment
-log10(pval): depletion
% enrichment
OPN
DPN
TATA
Tless
responsive
non-responsive
essential
periodic
non-essential

Fig. S4

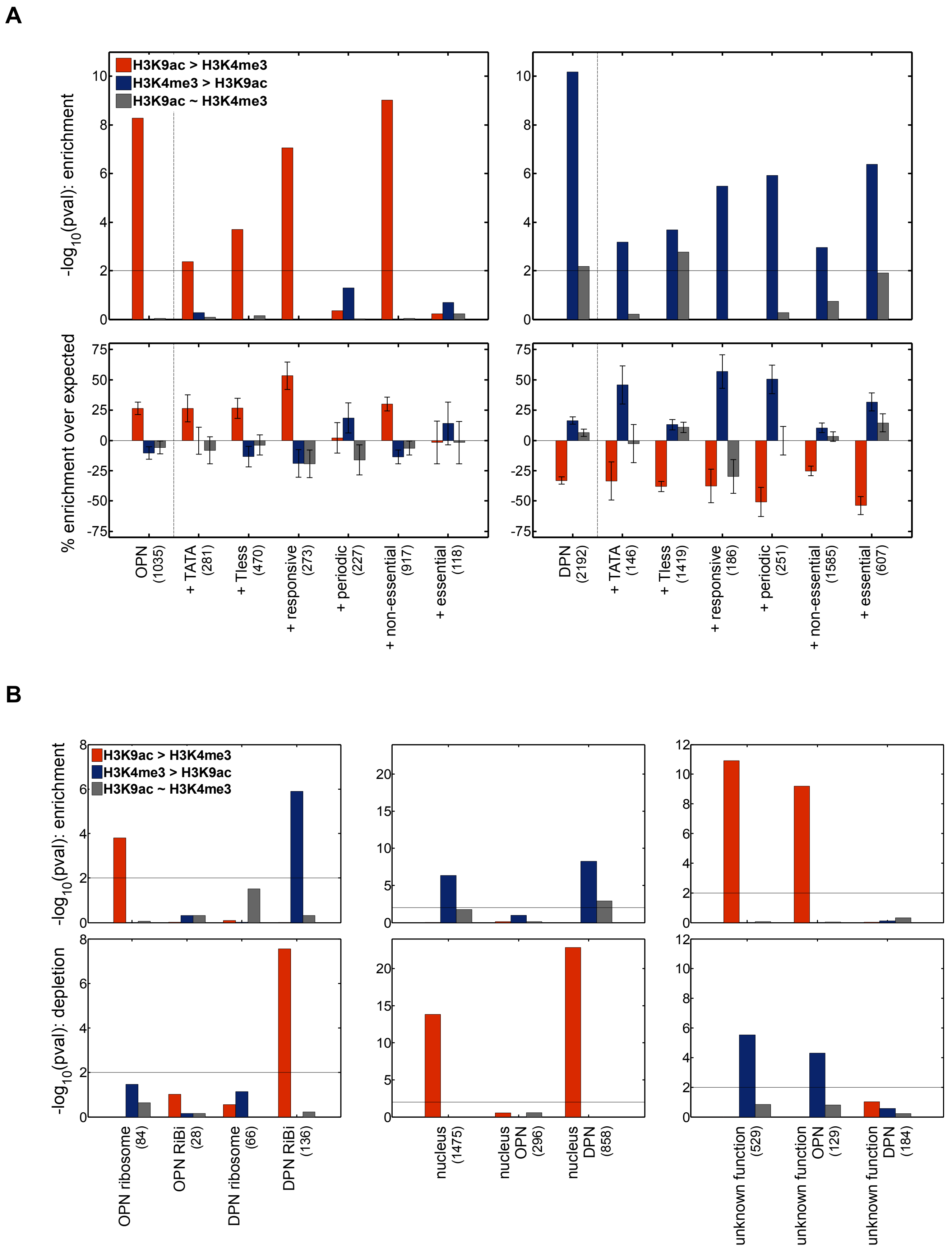

A
H3K9ac > H3K4me3
H3K4me3 > H3K9ac
H3K9ac ~ H3K4me3
$-log_{10}$(pval): enrichment
% enrichment over expected
OPN (1035)
+ TATA (281)
+ Tless (470)
+ responsive (273)
+ periodic (227)
+ non-essential (917)
+ essential (118)
DPN (2192)
+ TATA (146)
+ Tless (1419)
+ responsive (186)
+ periodic (251)
+ non-essential (1585)
+ essential (607)
B
H3K9ac > H3K4me3
H3K4me3 > H3K9ac
H3K9ac ~ H3K4me3
$-log_{10}$(pval): enrichment
$-log_{10}$(pval): depletion
OPN ribosome (84)
OPN RiBi (28)
DPN ribosome (66)
DPN RiBi (136)
nucleus (1475)
nucleus OPN (296)
nucleus DPN (858)
unknown function (529)
unknown function OPN (129)
unknown function DPN (184)

Fig. S5

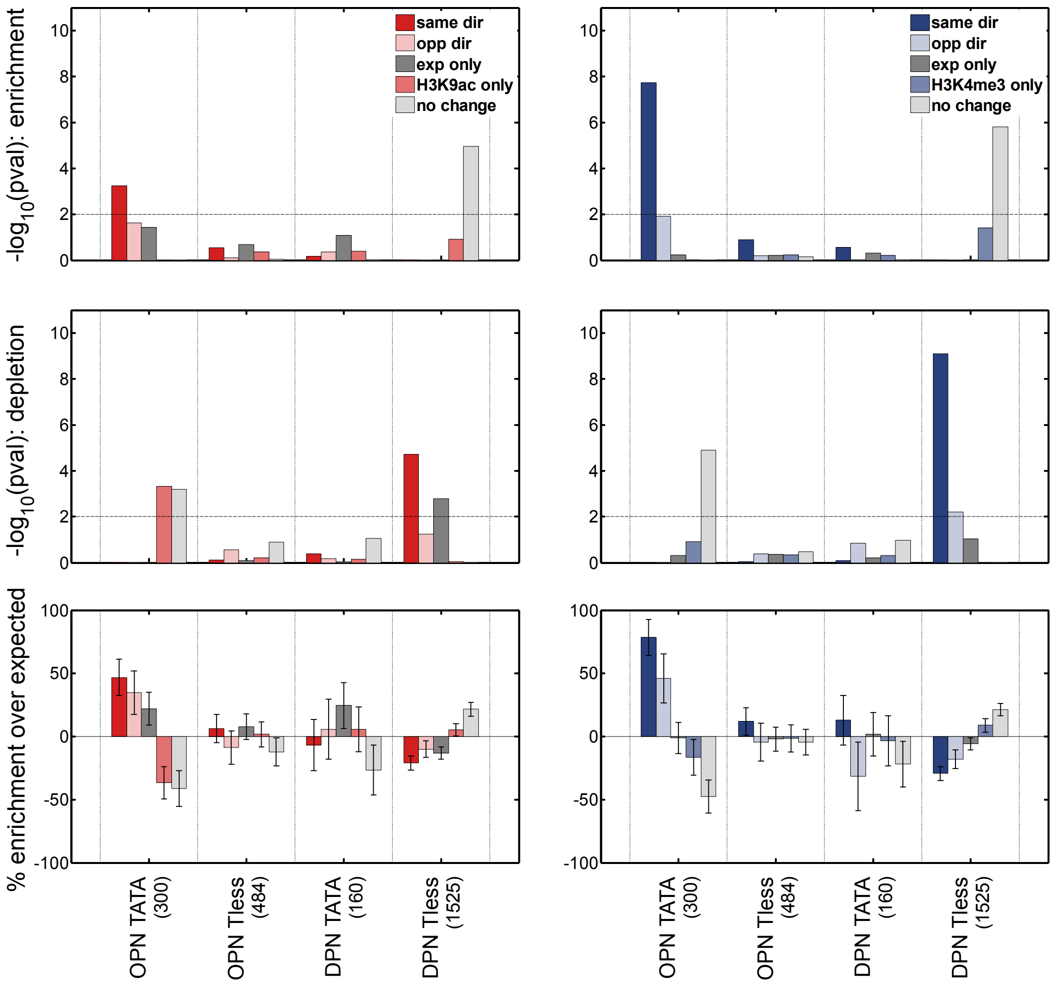

same dir
opp dir
exp only
H3K9ac only
no change
same dir
opp dir
exp only
H3K4me3 only
no change
-log10(pval): enrichment
-log10(pval): depletion
% enrichment over expected
OPN TATA (300)
OPN Tless (484)
DPN TATA (160)
DPN Tless (1525)

Fig. S6

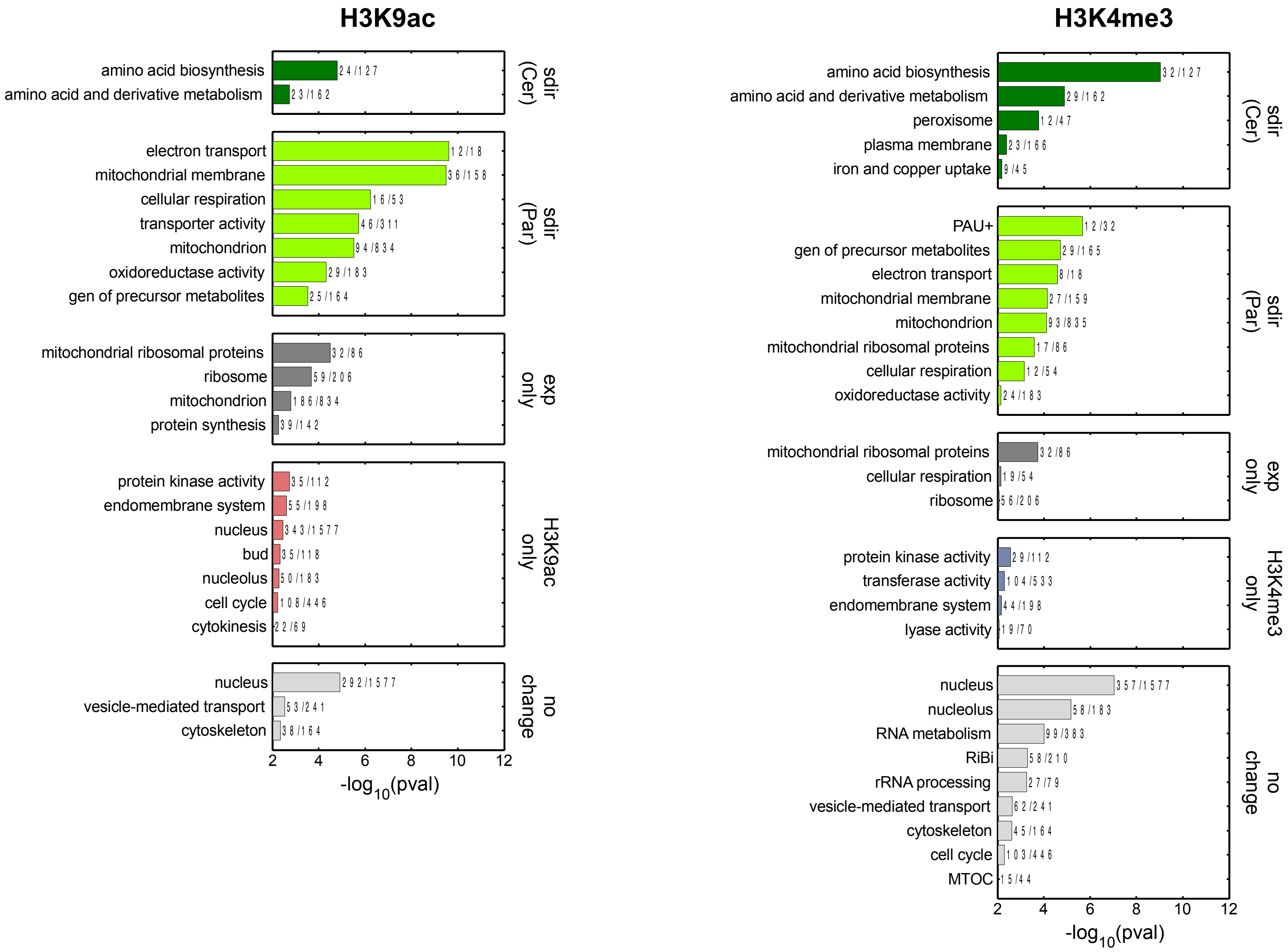
H3K9ac
amino acid biosynthesis
amino acid and derivative metabolism
24/127
23/162
sdir (Cer)
electron transport
mitochondrial membrane
cellular respiration
transporter activity
mitochondrion
oxidoreductase activity
gen of precursor metabolites
12/18
36/158
16/53
46/311
94/834
29/183
25/164
sdir (Par)
mitochondrial ribosomal proteins
ribosome
mitochondrion
protein synthesis
32/86
59/206
186/834
39/142
exp only
protein kinase activity
endomembrane system
nucleus
bud
nucleolus
cell cycle
cytokinesis
35/112
55/198
343/1577
35/118
50/183
108/446
22/69
H3K9ac only
nucleus
vesicle-mediated transport
cytoskeleton
292/1577
53/241
38/164
no change
2 4 6 8 10 12
-log10(pval)
H3K4me3
amino acid biosynthesis
amino acid and derivative metabolism
peroxisome
plasma membrane
iron and copper uptake
32/127
29/162
12/47
23/166
9/45
sdir (Cer)
PAU+
gen of precursor metabolites
electron transport
mitochondrial membrane
mitochondrion
mitochondrial ribosomal proteins
cellular respiration
oxidoreductase activity
12/32
29/165
8/18
27/159
93/835
17/86
12/54
24/183
sdir (Par)
mitochondrial ribosomal proteins
cellular respiration
ribosome
32/86
19/54
56/206
exp only
protein kinase activity
transferase activity
endomembrane system
lyase activity
29/112
104/533
44/198
19/70
H3K4me3 only
nucleus
nucleolus
RNA metabolism
RiBi
rRNA processing
vesicle-mediated transport
cytoskeleton
cell cycle
MTOC
357/1577
58/183
99/383
58/210
27/79
62/241
45/164
103/446
15/44
no change
2 4 6 8 10 12
-log10(pval)

Fig. S7
